# IL-17 signaling in primary sclerosing cholangitis patient-derived organoids

**DOI:** 10.1097/HC9.0000000000000454

**Published:** 2024-06-03

**Authors:** Ana S. Garcia Moreno, Maria E. Guicciardi, Alexander Q. Wixom, Erik Jessen, Jingchun Yang, Sumera I. Ilyas, Jackie K. Bianchi, Filippo Pinto e Vairo, Konstantinos N. Lazaridis, Gregory J. Gores

**Affiliations:** 1 Division of Gastroenterology and Hepatology, Department of Medicine, Mayo Clinic, Rochester, Minnesota, USA; 2 Department of Quantitative Health Sciences, Mayo Clinic, Rochester, Minnesota, USA; 3 Center for Individualized Medicine, Department of Medicine, Mayo Clinic, Rochester, Minnesota, USA; 4 Department of Clinical Genomics, Mayo Clinic, Rochester, Minnesota, USA

## Abstract

**Background:**

The pathogenesis of primary sclerosing cholangitis (PSC) is unclear, although studies implicate IL-17A as an inflammatory mediator in this disease. However, a direct assessment of IL-17 signaling in PSC cholangiocytes is lacking. In this study, we aimed to investigate and characterize the response of PSC extrahepatic cholangiocyte organoids (ECO) to IL-17A stimulation.

**Methods:**

Cholangiocytes obtained from patients with PSC and without PSC by endoscopic retrograde cholangiography were cultured as ECO. The ECO were treated with vehicle or IL-17A and assessed by transcriptomics, secretome analysis, and genome sequencing.

**Results:**

Unsupervised clustering of all integrated single-cell RNA sequencing data identified 8 cholangiocyte clusters that did not differ between PSC and non-PSC ECO. However, PSC ECO cells demonstrated a robust response to IL-17 treatment, as noted by an increased number of differentially expressed genes by transcriptomics and more abundant chemokine and cytokine expression and secretion. After rigorous filtering, genome sequencing identified candidate somatic variants shared among PSC ECO from unrelated individuals. However, no candidate rare variants in genes regulating the IL-17 pathway were identified, but rare variants regulating the MAPK signaling pathway were present in all PSC ECO.

**Conclusions:**

PSC and non-PSC patient-derived ECO respond differently to IL-17 stimulation, implicating this pathway in the pathogenesis of PSC.

## INTRODUCTION

Primary sclerosing cholangitis (PSC) is a heterogeneous, chronic cholestatic liver disease characterized by fibroinflammatory biliary tract strictures of the intrahepatic and extrahepatic bile ducts. The resultant fibroinflammatory process is usually progressive, and patients with PSC frequently advance to end-stage liver disease, necessitating liver transplantation to extend survival.^1^ Rational therapies for PSC are lacking and will require an understanding of its molecular and cellular pathogenesis, especially insight into the mechanisms causing persistent injury to cholangiocytes (the cells lining the bile ducts). Although regarded as an immune-mediated disease, the pathogenesis of PSC remains elusive, mainly due to difficulty in accessing cholangiocytes, the instability of in vitro monolayer culture systems of primary cells and the lack of animal models that reproducibly recapitulate the human disease.^2^,^3^ Over the last few years, a new cell-based model has been developed to address several of these deficiencies termed three-dimensional organoid culture systems. Cholangiocyte organoids permit primary human cells to self-organize through cell-cell and cell-matrix interactions and have proven to be a powerful tool in the study of development, disease pathogenesis, and regeneration of the liver.^4^,^5^ Primary cholangiocytes have been successfully obtained from bile and/or brushing collected during clinically indicated endoscopic retrograde cholangiography (ERC) procedures and grown into organoids.^2^,^6^,^7^,^8^ In PSC, these patient-derived cholangiocyte organoids retain immunoreactive characteristics associated with PSC and can be maintained long-term in vitro.^2^ Due to regional diversity within the human biliary tree, it is important to obtain cells from large bile ducts, which are involved in PSC.^9^,^10^ Indeed, although PSC is characterized by inflammatory damage to both the large and small bile ducts of the liver, a pathognomonic feature of PSC is stricturing and obstruction of the large bile ducts as visualized by cholangiography.^11^ For example, noninvasive magnetic resonance cholangiography is now the diagnostic approach of choice for making a diagnosis of PSC and may be strikingly abnormal despite minimal parenchymal liver disease.^12^ Although the large bile ducts are, therefore, a specific target of this disease, little is known about the biology of these cholangiocytes in general and in PSC. The availability of human disease-derived extrahepatic cholangiocyte organoids (termed ECO by consensus),^5^ permits the interrogation of disease alterations afflicting cholangiocytes lining the large bile ducts in PSC.

The role of the IL-17 signaling pathway has been described in several diseases, including those with an immune etiology, and this pathway has been successfully targeted therapeutically in human diseases such as psoriasis and ankylosing spondylitis.^13^,^14^ The IL-17 family of ligands comprises 6 members, IL-17A to IL-17F, along with its corresponding 5-member receptor family (IL17RA-IL17RE).^15^ Notably, the presence of receptors A and C within this family has been identified in human cholangiocytes, suggesting a potential involvement of IL-17 signaling in cholangiocyte-related processes.^16^ IL-17A is prominently expressed in human diseases and has been broadly studied, therefore it is considered to be the main inducer of the IL-17 signaling pathway.^17^,^18^ It shares a great percentage of conservation with IL-17F and it is produced predominantly by a T-cell subset termed Th17 cells.^18^,^19^ IL-17A can be secreted by other cell types, including γδ T-cells, mucosal-associated invariant T cells, CD8^+^ T-cells and neutrophils in the liver.^20^,^21^,^22^ Th17 cells and IL-17^+^ CD8^+^ T-cells are considered, however, the major source of IL-17 in several inflammatory liver diseases.^23^ Interestingly, evidence in other liver diseases, such as alcohol-associated liver disease and viral hepatitis C, suggest a correlation of IL-17A expression and liver fibrosis.^24^ In PSC, recent human studies have proficiently identified IL-17A within liver tissue sections, demonstrating a discernible increase in IL-17–producing cells in periductal regions among patients with PSC when compared to control subjects^25^,^26^,^27^ and compelling findings suggest that secretion of IL-17A by peripheral blood mononuclear cells (PBMCs), is elevated in individuals with PSC compared to those with other inflammatory liver diseases.^26^ Moreover, PBMCs from patients with PSC display an augmented Th17 cell response to pathogens in vitro.^25^ This aligns with the outcomes of genome-wide association studies, which have precisely identified polymorphisms in genes linked to the generation of Th17 cells.^28^,^29^

Correspondingly, *Mdr2*^*−/−*^ and bile duct ligated mice, both models of cholestatic liver injury, demonstrate an increase in expression of hepatic IL-17A and its receptor IL-17RA, and aggregation of IL-17–producing cells in periductal areas.^24^,^30^ In addition, other studies have reported a decrease in hepatic neutrophil accumulation, liver fibrosis, and liver damage in IL-17A or IL-17RA knockout mice or by blocking IL-17A.^24^,^31^,^32^,^33^,^34^ These studies suggest an injurious role for IL-17 signaling in cholangiocytes and cholestatic liver injury. However, a recent study in mice cholangiocyte organoids determined that IL-17 induces programmed cell death ligand-1 expression in cholangiocytes^27^; induction of programmed cell death ligand-1 would be expected to impair T-cell activation and limit T-cell–mediated liver injury. Hence, the role of IL-17A in cholestatic liver injury is complex and requires further definition.

Recently, a study involving patients with concomitant PSC and inflammatory bowel disease (IBD) reported a transcriptional signature associated with an increased risk of colon dysplasia that is characterized by a pathogenic IL-17 signature in T cells.^35^ In addition, studies conducted on colon organoids from patients with IBD identified somatic mutations, which dysregulate the IL-17 signaling pathway.^36^ These observations are quite pertinent to PSC, as the majority of patients with PSC have IBD, and parallels exist in the mucosal injury of the colon and bile ducts in these diseases. However, information regarding somatic mutations and/or dysregulated IL-17 signaling in PSC cholangiocytes is lacking. Therefore, our aims were to characterize the response of PSC-derived ECO to IL-17, with the goal of determining if IL-17 signaling is differentially regulated in PSC versus non-PSC cholangiocytes.

## METHODS

### Patient enrollment

Patients with PSC were diagnosed using criteria established by the American Association for the Study of Liver Disease (AASLD) guidelines.^37^ Patients, with PSC or without PSC, who were undergoing clinically indicated ERC, were identified through the electronic medical record (Table 1). Patients with known malignancy, OLT, a history of biliary-enteric anastomosis, or other chronic liver diseases were excluded from this study. The study, including sample collection, was approved by the Mayo Clinic Institutional Review Board, all research was performed in accordance with both the Declarations of Helsinki and Istanbul, and written informed consent was obtained from all subjects and/or their legal guardian(s) prior to ERC.

**TABLE 1:** Baseline characteristics of the patients included in the study at the time of ERC

Patient	Diagnosis	Sex	Age	IBD	IBD type	Cirrhosis	Indication for ERC	Anatomical location of sample	T Bili (mg/dL)	Alk Phos (92–279 IU/L)
1	PSC, AIH	F	68	No	—	Yes	Stent removal	Common hepatic duct, right main duct	3.7	583
2	PSC	F	48	Yes	UC	Yes	Elevated bilirrubin	Distal common bile duct	2.6	37
3	PSC	M	77	No	—	No	Stent removal left hepatic duct	Left main duct	10.1	394
4	PSC	F	70	Yes	UC	No	Right hepatic duct stricture	Common bile duct, common hepatic duct	0.3	159
5	PSC	F	34	Yes	UC	No	Follow-up (? cystic duct malignancy)	Bile	—	207
6	PSC	M	56	Yes	UC	Yes	Elevated bilirubin	Common bile duct	6.6	552
7	PSC	F	60	Yes	CD	No	Follow-up (mass on MRI segment 4)	Bile	0.6	58
8	PSC	M	30	Yes	UC	No	Surveillance	Bile	1.7	210
9	PSC	M	63	Yes	UC	Yes	Biliary stent removal	Left main duct	2.8	408
10	PSC	M	60	No	—	Yes	Surveillance	Common hepatic duct	1.7	967
1	Non-PSC	M	65	No	—	No	Bile leak, benign biliary stricture (Stent removal or exchange)	Common bile duct bifurcation	0.6	158
2	Non-PSC	M	72	No	—	No	Benign biliary stricture with lab abnormalities	Distal common bile duct	—	279
3	Non-PSC	M	80	No	—	No	Pancreatic and biliary stricture	Common bile duct	1.1	76
4	Non-PSC	M	69	No	—	No	Chronic pancreatitis, stent change	Common bile duct	0.3	103
5	Non-PSC	F	54	No	—	No	Abdominal pain with persistent elevation of liver enzymes	Distal common bile duct	0.2	145
6	Non-PSC	F	62	No	—	No	Follow-up of bile leak	Common bile duct, Bile: cystic duct	0.7	126
7	Non-PSC	M	66	No	—	No	Biliary stent removal	Distal common bile duct	1.1	137

*Note:* Presence of cirrhosis was determined by one or more of the following diagnostic techniques: elastography, cross-sectional imaging with cirrhotic liver morphology, evidence of portal hypertension, and/or liver biopsy.

Abbreviations: AIH, autoimmune hepatitis; CD, Crohn disease; ERC, endoscopic retrograde cholangiography; IBD, inflammatory bowel disease; PSC, primary sclerosing cholangitis; UC, ulcerative colitis.

### Tissue collection

Following specific cannulation of the common bile duct, bile was aspirated via a catheter. Up to 10 mL of bile and/or the brush following cytology of extrahepatic bile ducts were collected and placed on ice prior to processing. Once processed, the samples were used for ECO generation within the first hour after being collected. As noted in Table 1, 7 of the 10 PSC and all the control samples were acquired through brushing. Hence, the vast majority of the samples were from the extrahepatic biliary tree. Only one of the organoids was subjected to single-cell RNA sequencing (scRNA-seq) and NanoString analysis, and 2 from the Olink were from bile, whereas all the others were from brush samples. We also note that a recent publication demonstrated that organoid-specific and regional gene profiles indicate that bile-derived organoids are of extrahepatic origin.^38^ Importantly, the entire biliary system, including intrahepatic biliary cells, are involved in PSC pathogenesis.

### Organoid generation and culture

Bile samples were diluted 1:10 with ice-cold PBS + anti-anti (1:100 dilution) and processed as described by Soroka et al.^2^ The cytology brush was placed in a sterile polystyrene petri dish containing PBS + anti-anti (1:100 dilution), and a sterile pipette was used to mechanically remove the cellular material embedded in the brush. The sample was transferred to a 15-mL conical tube and centrifuged at 300*g* for 5 minutes, then the supernatant was removed, and the resulting pellet was washed twice with ice-cold PBS + anti-anti and once with advanced DMEM/F12. The cell pellets from both brushing and bile samples were resuspended in Matrigel, dispensed onto 48-well plates as 30-μL droplets, and grown in an organoid complete medium.^2^ After 3 days, the organoid expansion medium defined by Soroka et al^2^ was employed to expand the organoids and was refreshed every 2 days; ECO were passaged when ~75% confluent with a split ratio of 1:3/1:4 using Cell Recovery Solution on ice for 30 minutes. In further experiments, ECO were treated with vehicle or recombinant human IL-17A (100 ng/mL) for 24 hours, aligning with previous studies on cholangiocyte organoids.^27^ This enabled us to maintain consistency in the literature and facilitate comparisons. Additionally, the use of a higher dose aimed to compensate for the sequestration and the loss of IL-17A due to the use of Matrigel.^39^

Other material and methods are available in the Supplemental Materials, Supplemental Digital Content 1, http://links.lww.com/HC9/A900.

## RESULTS

### Characterization of human ECO

**FIGURE 1:**
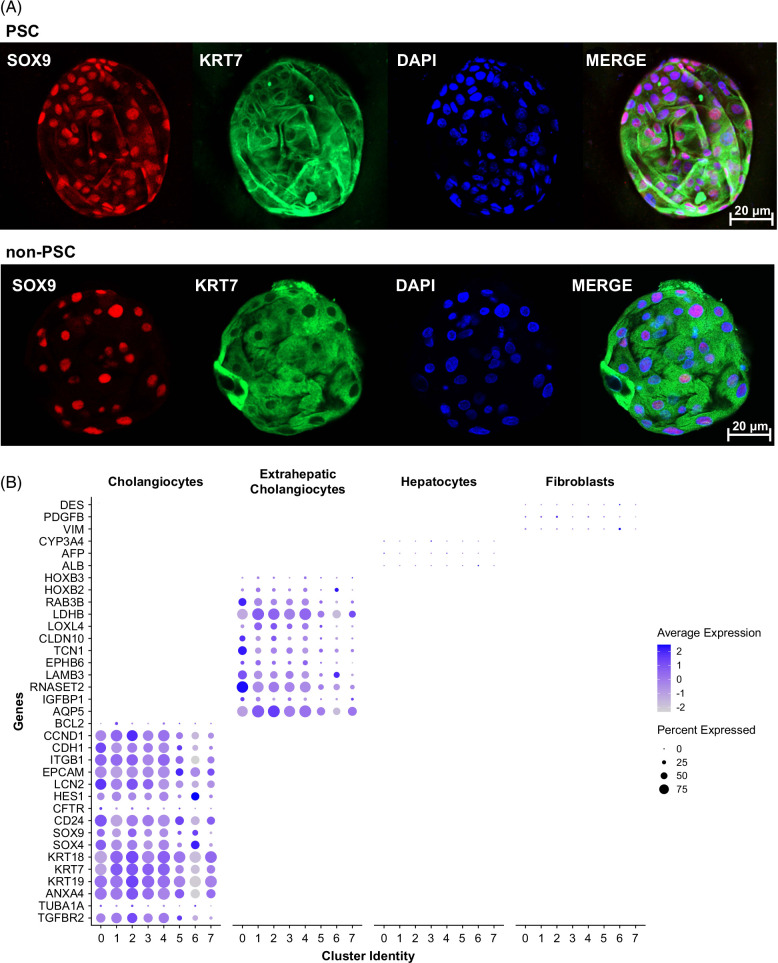
Characterization of patient-derived ECO. (A) Whole mount immunofluorescence of PSC and non-PSC ECO stained with SOX9 (red), KRT7 (green), and DAPI (blue) obtained by confocal (objective=×20, scale bar=20 µm). (B) Dot plot displaying expression of known classical cholangiocyte, extrahepatic bile duct, hepatocyte, and fibroblast marker genes in each cholangiocyte cluster identified by single cell RNA seq analysis of ECO. The intensity of the color indicates the average expression, and the size of the circle is directly proportional to the percentage of cells expressing each gene. Abbreviations: ECO, extrahepatic cholangiocyte organoid; KRT7, cytokeratin 7; PSC, primary sclerosing cholangitis; SOX 9, SRY-box transcription factor 9.

We obtained ECO from patients with PSC (n=9) and with PSC (n=7). Patient characteristics and indications for the ERC are described in Table 1. To confirm the cholangiocyte phenotype of the ECO, we initially performed whole-mount immunofluorescence for SRY-box transcription factor 9, and cytokeratin 7 (KRT7), both expressed uniquely in cholangiocytes within the liver. SOX 9 and KRT7 were expressed in both PSC and non-PSC–derived ECO and appeared to be expressed by all cells within the organoids (Figure 1A). Next, we performed scRNA-seq of the PSC (n=4) and non-PSC (n=4) patient-derived ECO. Cholangiocyte gene expression profiles were strongly enriched in all clusters (KRT7, KRT18, and KRT19; epithelial cellular adhesion molecule), whereas genes expressed in hepatocytes and/or fibroblasts but not cholangiocytes were not significantly expressed in any of the clusters (albumin, alpha-fetoprotein, cytochrome P450 family 3 subfamily A member 4, desmin, PDGF subunit B, and vimentin). Genes previously identified in ECO and extrahepatic bile ducts were also expressed by all clusters and did not demonstrate any differences between the anatomical regions of the extrahepatic bile ducts (homeobox B2 (HOXB2), homeobox B3 (HOXB3), aquaporin 5, insulin-like growth factor binding protein 1, ribonuclease T2, laminin subunit beta 3, and lactate dehydrogenase B) (Figure 1B).^2^,^9^,^10^ These observations verify the extrahepatic cholangiocyte phenotype of the cells within the ECO and are consistent with the observations of others.^9^,^10^

### Characterization of cholangiocyte genetic heterogeneity by scRNA-seq

**FIGURE 2:**
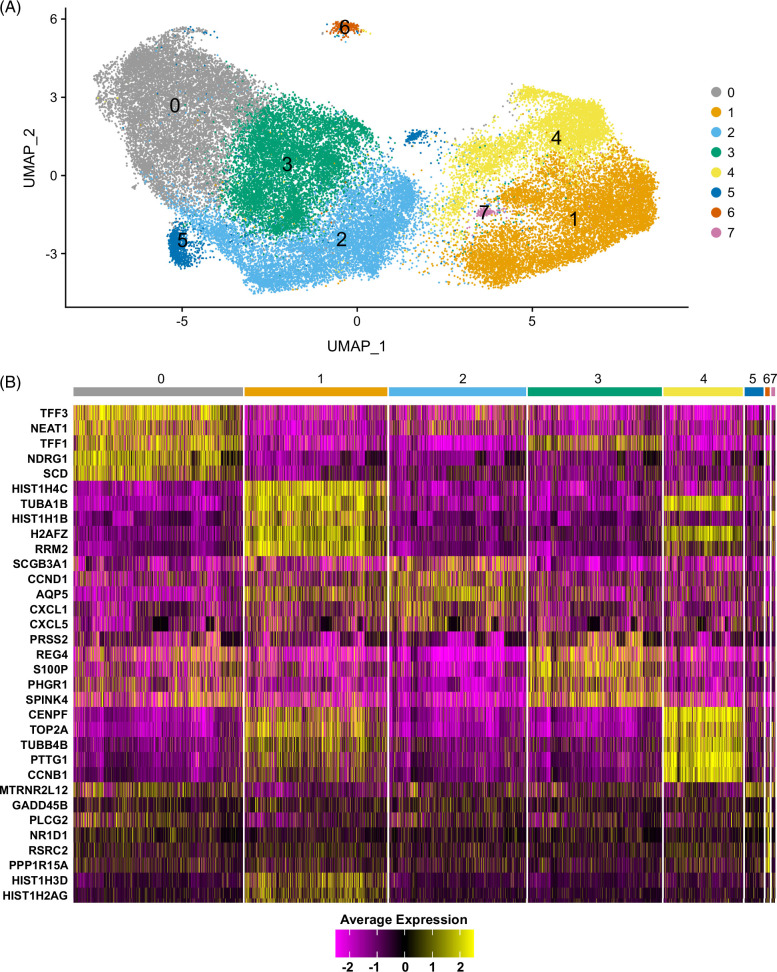
Identification of distinct cholangiocyte clusters in ECO. ECO from patients with primary sclerosing cholangitis (n=4) and without primary sclerosing cholangitis (n=4) were subjected to single-cell RNA seq. (A) UMAP plot visualizing 8 distinct clusters termed Cluster 0 to 7, obtained by unsupervised clustering. Colors represent a unique cell population cluster as identified by transcriptional signature. (B) Heatmap displaying the top 5 cluster marker genes for each unique cell population cluster. Colors indicate the expression of each gene in a cluster, when compared to all other cluster combined (yellow=upregulation, purple=downregulation). Abbreviation: ECO, extrahepatic cholangiocyte organoid.

Given the known genetic heterogeneity of cholangiocytes, we examined genetic markers in the PSC and non-PSC ECO. Interrogating scRNA-seq data, we identified 8 clusters shared between the ECO derived from all samples (Figure 2A, Supplemental Figure S1, Supplemental Digital Content 1, http://links.lww.com/HC9/A900). The top 5 conserved cluster marker genes are displayed as a heatmap in Figure 2B. Cluster 0 was characterized by an enhanced expression of genes associated with mucosal maintenance (TFF31, TFF2, and TFF3), hypoxia (NDRG1, EGLN3, and CA9), reactive oxygen species (ERO1A, DUOX2, and its maturation factor DUOXA2) and 2 long noncoding RNA genes (MALAT1 and NEAT1) (Figure 2B, Supplemental File S1, Supplemental Digital Content 2, http://links.lww.com/HC9/A895). Clusters 1 and 4 had increased expression of MKI67 and PCNA, suggesting an active proliferative state in these clusters at the time of the analysis (Supplemental File S1, Supplemental Digital Content 2, http://links.lww.com/HC9/A895). Interestingly, Cluster 2 expressed TNFSF15, TNFRSF12A, TNFAIP2, CXCL1, CXCL5, CXCL8, CCND1, and IL-18, consistent with an inflammatory phenotype^40^ (Figure 2B, Supplemental File S1, Supplemental Digital Content 2, http://links.lww.com/HC9/A895). Cluster 5 and Cluster 7 had a limited number of conserved cluster marker genes. Cluster 5 had only one conserved cluster marker gene, MT-RNR2 Like 12, which is a mitochondrial-derived peptide that exerts anti-apoptotic effects by preventing the translocation of Bax from the cytosol to mitochondria.^41^ Cluster 7 had 3 conserved markers, all of them belonging to histone coding genes (HIST1H1B, HIST1H3D, and HIST1H2AG) (Figure 2B, Supplemental File S1, Supplemental Digital Content 2, http://links.lww.com/HC9/A895). Lastly, Cluster 6 had increased expression of genes associated with cell damage, such as GADD45B, GADD45G, PPP1R15A, and HSPB1 (Figure 2B, Supplemental File S1, Supplemental Digital Content 2, http://links.lww.com/HC9/A895), which likely indicates that the cells in this cluster are manifesting a stress response. Altogether, the identification of different cell clusters confirms the heterogeneity of extrahepatic cholangiocytes.

### Transcriptomic profiling of non-PSC and PSC ECO demonstrate differences between the 2 groups

**FIGURE 3:**
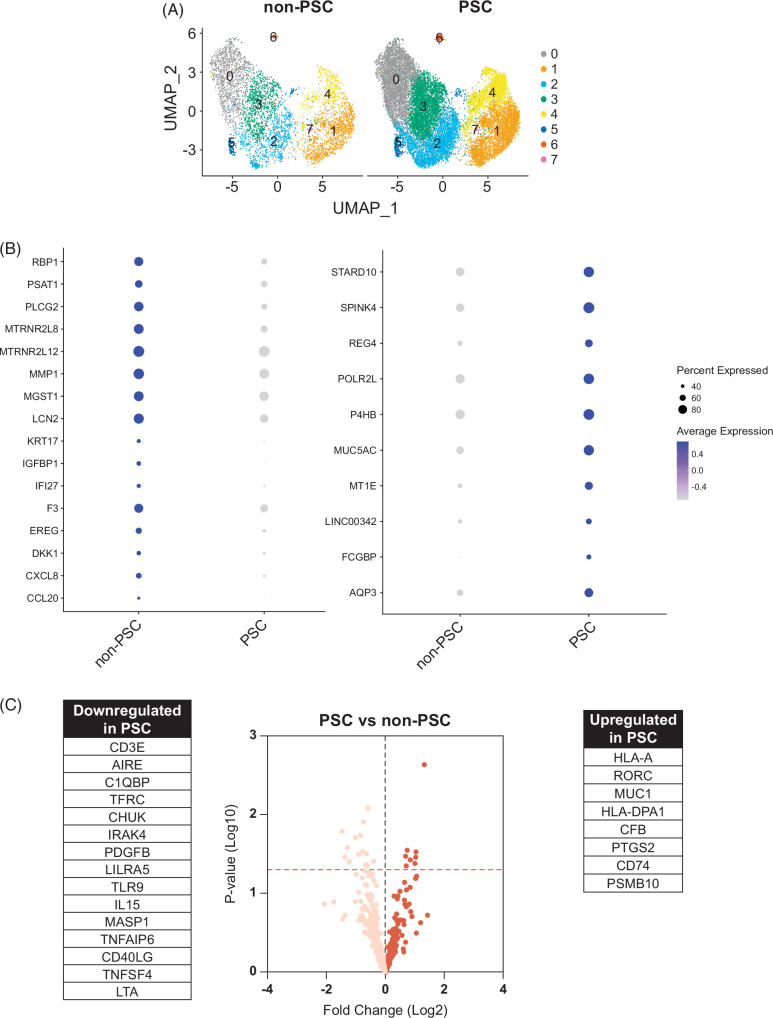
Transcriptomic profiling of PSC and non-PSC ECO. (A) UMAP plots comparing clusters from PSC (n=4) and non-PSC (n=4) ECO. (B) Dot plots visualizing differentially expressed genes between PSC and non-PSC ECO. Color indicates average expression; size of the circle indicates percentage of cells expressing each gene. Differentially expressed genes were determined by a *p*-value <0.05, Log2Fold change >0.2, and their presence in at least 4 clusters. (C) Volcano plot comparing PSC (n=6) and non-PSC (n=5) ECO by NanoString analysis. Dots represent a downregulated (left) or upregulated (right/highlighted) gene. Line (red) indicates *p*=0.05. Statistically significant genes are listed next to volcano plot. Abbreviations: ECO, extrahepatic cholangiocyte organoid; PSC, primary sclerosing cholangitis.

Once the clusters were identified and characterized, we elected to investigate whether a cluster or multiple clusters were different between PSC and non-PSC ECO by comparing the cell percentage of each cluster. However, no significant differences were identified (Figure 3A and Supplemental Figure S2, Supplemental Digital Content 1, http://links.lww.com/HC9/A900). These data suggest that PSC ECO do not have a unique and characteristic cell population of cholangiocytes when compared to non-PSC ECO but rather share the same cholangiocyte populations. Nonetheless, there can be differences in expression of genes shared among clusters that do not distinguish individual clusters per se but yet differ between PSC and non-PSC ECO. Therefore, we analyzed the transcriptional profiles of the groups by examining differentially expressed genes (DEGs) between PSC and non-PSC ECO. DEG analysis led to the identification of genes that were consistently enriched in PSC ECO and in non-PSC ECO in the majority of the clusters. The main enriched genes in PSC ECO were found to be AQP3, FCGBP, LINC00342, MT1E, MUC5AC, P4HB, POLR2L, PPIB, REG4, SPINK4, and STARD10 (Figure 3B, Supplemental Figure S2, Supplemental Digital Content 1, http://links.lww.com/HC9/A900). On the other hand, non-PSC ECO demonstrated enrichment of C-C motif chemokine ligand (CCL)20, CXCL8, DKK1, EREG, F3, IFI27, insulin-like growth factor binding protein 1, KRT17, LCN2, MGST1, MMP1, MT-RNR2 Like 12, MTRNR2L8, PLCG2, PSAT1, and RBP1 (Figure 3B, Supplemental Figure S2, Supplemental Digital Content 1, http://links.lww.com/HC9/A900). These data confirm that differences in gene expression exist throughout cholangiocyte populations when comparing PSC and non-PSC ECO.

We additionally elected to investigate the differences in the expression of inflammation-related genes between PSC and non-PSC ECO by NanoString analysis, given that different methodologies yield complementary results. These data demonstrated that PSC ECO have a higher expression of HLA-A, RORC, MUC1, HLA-DPA1, CFB, PTGS2, CD74, and PSMB10 when compared to non-PSC (Figure 3C). Downregulated genes in PSC ECO included CD3E, AIRE, C1QBP, TFRC, and CHUK (Figure 3C). Furthermore, we then selected statistically significant DEG and performed functional annotation analysis; these indicated the enrichment of pathways associated with both innate and adaptative immune responses and were consistent with liver diseases such as cholestasis and metabolic dysfunction-associated fatty liver disease (Supplemental Figure S3, Supplemental Digital Content 1, http://links.lww.com/HC9/A900). These results indicate a baseline difference in gene expression between PSC and non-PSC ECO and their respective cholangiocyte populations. Interestingly, both PSC and non-PSC ECO express inflammation-associated genes, but the specific genes and functions differ between the 2 groups.

### Secretome analysis reveals increased secretion of proinflammatory proteins by PCS ECO

To further understand the differences between PSC and non-PSC ECO at baseline, we investigated secreted inflammation-related proteins by performing Olink analysis on the supernatant of the ECO. Although both PSC and non-PSC ECO secreted inflammatory proteins, PSC ECO had a significantly higher release of proteins that included cytokines and chemokines such as IL-6, TRAIL, CXCL9, IL-2, CCL4, MCP-4, TNFSF14, IL-13, MCP-2, CCL25, and IL-5 (Supplemental Figure S4, Supplemental Digital Content 1, http://links.lww.com/HC9/A900). These data indicate that PSC and non-PSC ECO secrete inflammatory proteins; however, the secreted protein abundance for most of these proteins was greater in PSC ECO.

### PSC and non-PSC patient-derived ECO respond differently to IL-17A stimulation

**FIGURE 4:**
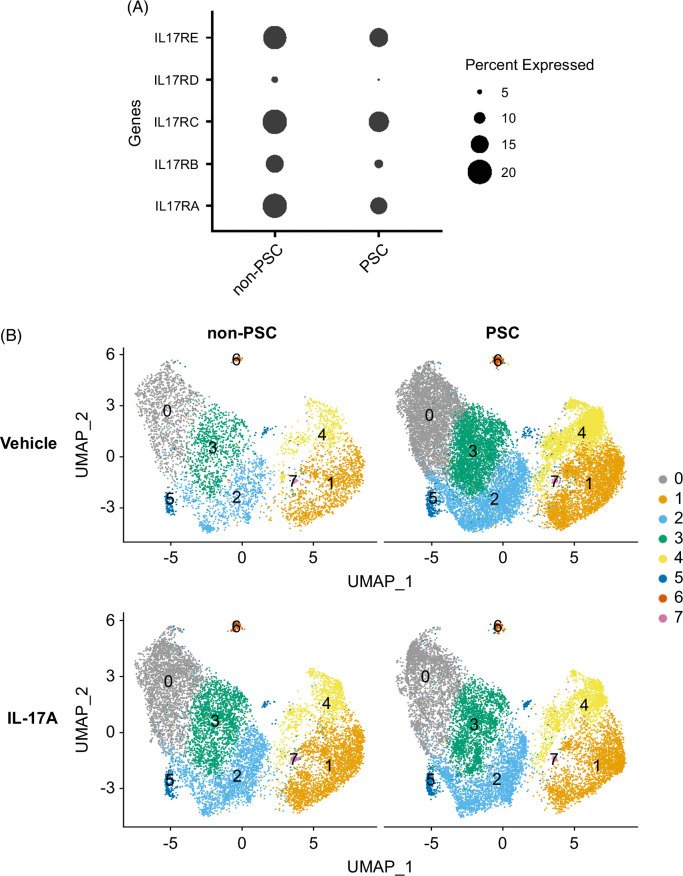
IL-17 response in PSC and non-PSC ECO. PSC and non-PSC extrahepatic cholangiocyte organoids were treated with vehicle or IL-17A (100 ng/mL) for 24 hours and subjected to single-cell RNA sequencing. (A) Dot plot displaying IL-17 receptor family expression represented as a pseudo bulk analysis. The size of the circle indicates the percentage of cells expressing each gene. (B) UMAP plots visualizing the different clusters in PSC and non-PSC, and treatments (vehicle and IL-17A). Colors represent unique cell population clusters as identified by transcriptional signature. Abbreviations: ECO, extrahepatic cholangiocyte organoid; PSC, primary sclerosing cholangitis.

The initial step during the IL-17 signaling pathway requires the binding of the IL-17 ligand family to its cognate receptors.^17^ Therefore, to further characterize the ECO and ensure that cholangiocytes expressed the requisite receptor(s) for ligand binding, we analyzed such expression on scRNA-seq data as a pseudo bulk analysis. Both non-PSC and PSC ECO demonstrated a similar expression of the receptor family, with ILRA, ILRC, and IL17RE being more abundantly expressed. Hence, both PSC and non-PSC patient-derived ECO express the requisite cognate receptor subunits to initiate IL-17A signaling (Figure 4A).

To define the direct effects of IL-17A treatment in PSC and non-PSC ECO, the ECO were stimulated with IL-17A, and scRNA-seq was performed. Initially, the cell percentage of each cluster in PSC and non-PSC ECO was evaluated after the treatment. However, no significant differences were identified within samples (Figure 4B, Supplemental Figure S2, Supplemental Digital Content 1, http://links.lww.com/HC9/A900). Hence, IL-17 treatment does not induce changes in cell ratios in different cholangiocyte cluster populations when comparing non-PSC and PSC ECO.

**FIGURE 5:**
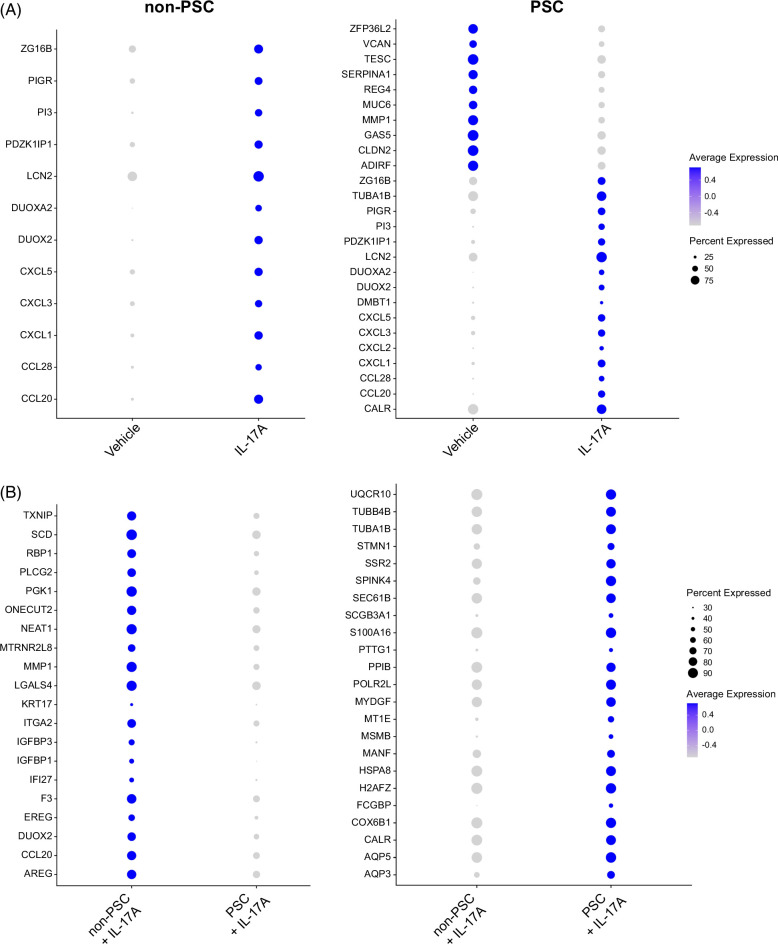
Differential response to IL-17A stimulation between non-PSC and PSC ECO by scRNA-seq. (A) Dot plots displaying pseudo bulk differentially expressed genes between ECO treated with vehicle and IL-17A within each condition (Vehicle vs IL-17A). (B) Dot plots displaying DEG between PSC and non-PSC ECO treated with IL-17A. The intensity of the color indicates the average expression, and the size of the circle indicates the percentage of cells expressing each gene. Differentially expressed genes were determined by a *p*-value<0.05 and Log2Fold change >0.2, and their presence in at least 4 clusters. Abbreviation: ECO, extrahepatic cholangiocyte organoids; PSC, primary sclerosing cholangitis.

Next, the DEG between vehicle and IL-17A-treated ECO within each group (PSC ECO ± IL-17A, non-PSC ECO ± IL-17A) were investigated, enabling the identification of genes that are either upregulated or downregulated by treatment in ECO. Both PSC and non-PSC ECO shared a common response to IL-17A, displaying changes in expression of CCL20, CCL28, CXCL1, CXCL3, CXCL5, DUOX2, DUOXA2, LCN2, PDZK1IP1, PI3, PIGR, and ZG16B (Figure 5A). However, there were also differences in genetic expression between the 2 cohorts. In particular, PSC ECO had an increased number of DEG after the treatment with IL-17A, and a significant number of these genes did not display expression changes in the non-PSC ECO (Figure 5A). These results imply that genetic regulation by IL-17A is different between non-PSC and PSC ECO.

To further understand the differences between PSC and non-PSC ECO, IL-17A treated ECO only (IL-17A treated PSC vs. IL-17A treated non-PSC ECO) were selected, and DEGs were investigated between these 2 cohorts. In this instance, the analysis enabled the identification of genes that are significantly different between PSC and non-PSC after treatment with IL-17A. PSC ECO displayed enrichment of aquaporin 5 (AQP5), CALR, COX6B1, H2AFZ, HSPA8, MANF, MSMB, MYDGF, PTTG1, S100A16, SCGB3A1, SEC61B, SSR2, STMN1, TUBA1B, TUBB4B, and UQCR10 (Figure 5B). Similarly, non-PSC ECO displayed the enrichment of AREG, DUOX2, IGFBP3, ITGA2, LGALS4, NEAT1, ONECUT2, PGK1, SCD, and TXMP (Figure 5B). Additional results describing DEGs by cluster are located in the Supplemental Files S3-S5. These results suggest that PSC ECO responds differently to IL-17A stimulation compared to non-PSC ECO.

**FIGURE 6:**
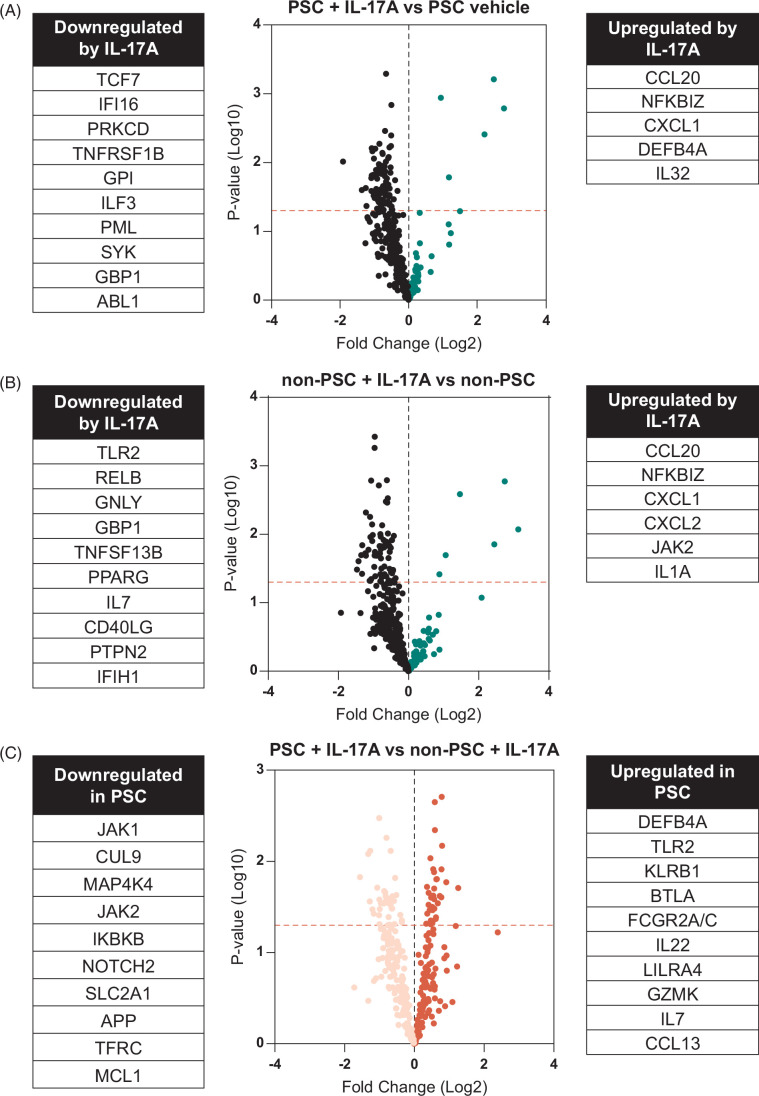
IL-17A response throughout distinct cholangiocyte cluster populations in non-PSC and PSC ECO. Volcano plots displaying mRNA expression profiles obtained by NanoString analysis comparing (A) vehicle-treated versus IL-17A-treated PSC ECO, (B) vehicle-treated versus IL-17A–treated non-PSC ECO (upregulated genes in green, downregulated genes in black), (C) IL-17A-treated PSC versus non-PSC ECO. Each dot represents a gene. Upregulated and downregulated genes are listed next to the volcano plots. Abbreviations: CCL, C-C motif chemokine ligand; ECO, extrahepatic cholangiocyte organoids; PSC, primary sclerosing cholangitis.

In addition to scRNA-seq, we again performed NanoString analysis and investigated inflammation-related genes after treatment with IL-17A. Initially, we investigated the effects of the treatment by comparing vehicle and IL-17–treated ECO. This confirmed the upregulation of CCL20 and CXCL1 (Figure 6A, B) in both ECO. In particular, PSC ECO displayed upregulation of DEFB4A and IL-32, which was not present in non-PSC ECO, making the upregulation of these genes unique to PSC (Figure 6A). Similarly, non-PSC ECO displayed upregulation of JAK2 and IL-1A after treatment with IL-17A (Figure 6B). Interestingly, IL-17 treatment appears to downregulate more than 30 genes in both ECO. Thus, we again selected statistically significant DEG and investigated the functional annotations associated with IL-17A stimulation within each ECO group. This analysis demonstrated differences in IL-17A regulation between non-PSC and PSC ECO, which is noted by the identification of pathways such as coronavirus pathogenesis, PKR in interferon induction, hepatic fibrosis, and IL-13 signaling in PSC ECO only (Supplemental Figure S3, Supplemental Digital Content 1, http://links.lww.com/HC9/A900). Given the importance of the IL-17A in liver fibrosis^42^ and the finding of the hepatic fibrosis signaling pathway mentioned above, we elected to investigate changes in expression of known markers of cholangiocyte-driven fibrosis genes in our scRNA-seq; however, none of these genes were significantly different between the samples (Supplemental Figure S5, Supplemental Digital Content 1, http://links.lww.com/HC9/A900).^43^,^44^ This suggests that fibrosis in PSC is likely multifactorial and perhaps not driven by IL-17A signaling in cholangiocytes itself but by other factors in the microenvironment. Also, a direct effect of IL-17A in the activation of HSCs, which secrete collagen, has been reported.^42^

When comparing IL-17 treated cells, PSC ECO expressed upregulation of inflammation-related genes such as DEFB4A, TLR2, KLRB1, BTLA, FCGR2A/C and IL22, LILRA4, GZMK, IL7, and CCL13 (Figure 6C). In a similar manner, PSC ECO expressed downregulation of JAK1, CUL9, MAP4K4, JAK2, and IKBKB (Figure 6C). Functional annotations of the genes mentioned above indicate a link between IL-17A stimulation in PSC ECO and the role of macrophages, fibroblasts, and endothelial cells in rheumatoid arthritis, natural killer cell and IL-6 signaling, and apoptosis. (Supplemental Figure S3, Supplemental Digital Content 1, http://links.lww.com/HC9/A900). Lastly, we performed Olink analysis on the supernatant of both PSC and non-PSC ECO to identify changes in secreted proteins. When comparing treated cells only, PSC ECO appears to have a higher secretion of various cytokines and chemokines such as MCP-3, IL7, CXCL11, CXCL9, CCL11, IL-10, TNF, CXCL6, IFN-gamma, CCL25, TWEAK, IL5, and TNFB (Supplemental Figure S6, Supplemental Digital Content 1, http://links.lww.com/HC9/A900).

Taken together, these results imply that the response to IL-17A is different between PSC and non-PSC ECO at the RNA and protein level, suggesting a role for this signaling pathway in PSC pathogenesis.

### Somatic variants in PSC ECO

**FIGURE 7:**
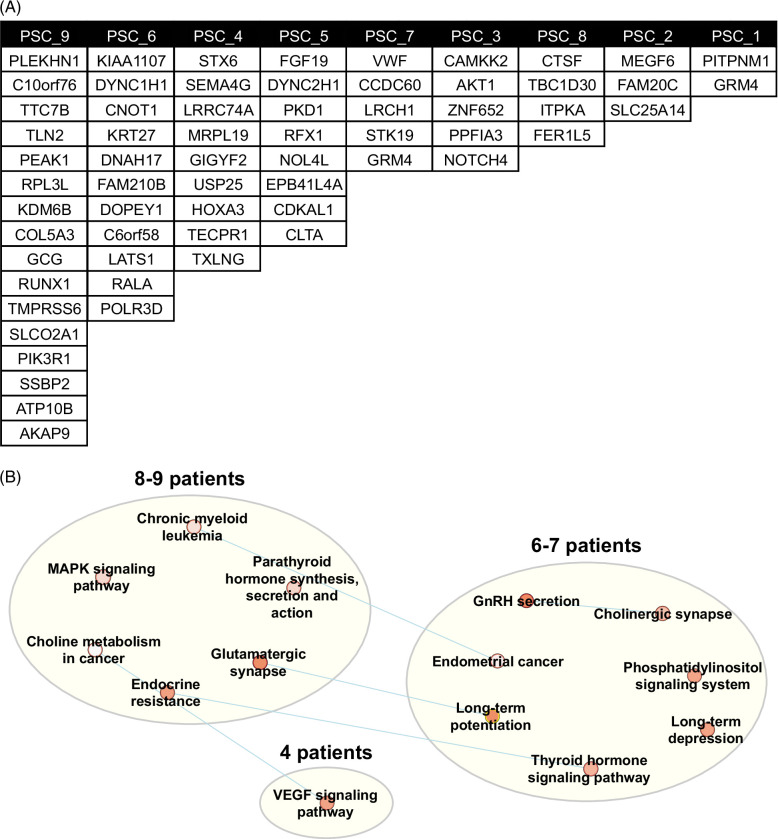
Identification of rare somatic variants in PSC ECO. WGS was performed in 9 PSC patient-derived ECO. (A) Table displaying somatic variants with CADD>25 by PSC ECO. (B) Somatic variant enriched pathways network of PSC ECO. Relaxed variant pathway analysis via Cytoscape and AutoAnnotate. Patients with variants in similar pathways are grouped. Each pathway is represented by a dot with a color indicating significance: white (*p*<0.05) to red (*p*<0.0001). Blue lines indicate pathways that share genes that contain a somatic variant. Abbreviation: ECO, extrahepatic cholangiocyte organoids; PSC, primary sclerosing cholangitis.

To investigate whether the differences in response to IL-17A between the ECO could be linked to a specific mutational signature, we examined somatic mutations based on the previously published work on ulcerative colitis and the IL-17 signaling pathway in the PSC ECO.^36^ Somatic variants were found by excluding confirmed germline variants in exome sequencing from peripheral white blood cells. Potentially deleterious variants, with a Combined Annotation-Dependent Depletion score^45^ higher than 25, were identified in all the patients, with a number of variants ranging from 2 to 16 (Figure 7A). However, none of the rare variants were in genes directly associated with the IL-17A signaling pathway. Acknowledging the fact that filtering in only variants with a high Combined Annotation-Dependent Depletion score excludes somatic variants that might have an impact on the protein, we relaxed the filtering criteria (Supplemental Figure S7, Supplemental Digital Content 1, http://links.lww.com/HC9/A900) and performed KEGG pathway analysis.^46^ Each of the patients had one or more rare somatic variants within at least 1 gene in several enriched pathways (Figure 7B). However, variants within the MAPK signaling pathway were driving the enrichment of these pathways and are depicted in Supplemental Figure S8, Supplemental Digital Content 1, http://links.lww.com/HC9/A900 and Supplemental Table S1, Supplemental Digital Content 1, http://links.lww.com/HC9/A900. Taken together, these data confirm the presence of somatic variants in PSC organoids. However, none of the rare variants were in genes related to the IL-17 signaling pathway. Of note, a limitation of this analysis is in comparing genome sequencing in the ECO to exome sequencing in PWBC.

## DISCUSSION

The results of this study provide insight into the differential signaling of IL-17A in ECO derived from patients with PSC. The approach of this study is summarized in Supplemental Figure S9, Supplemental Digital Content 1, http://links.lww.com/HC9/A900. The major and significant findings of this study are as follows: (1) both non-PSC and PSC ECO demonstrated similar cholangiocyte heterogeneity; (2) ECO derived from patients with PSC and without PSC both express IL-17 receptors A, C, and E; (3) ECO from PSC and non-PSC patients respond differentially to IL-17 stimulation with different gene expression and secretome; (4) no rare somatic variants were identified in genes associated with the IL-17A signaling pathway in PSC ECO, although rare somatic variants regulating the MAPK signaling pathway were identified. Taken together, the data suggest IL-17A signals differently in PSC versus non-PSC patient-derived cholangiocytes. These results are described in further detail below.

To get further insight into the PSC and non-PSC ECO in an unbiased manner and to characterize them in-depth, we applied scRNA-seq technology. This allowed us to examine cholangiocyte heterogeneity and led to the identification of 8 different clusters. However, the clusters were not different between PSC and non-PSC ECO, and neither did the cluster number or cell percentage of each cluster change with IL-17A stimulation. In another human cholangiopathy termed primary biliary cholangitis (PBC), a select population of cholangiocytes identified by the expression of DUOX and ACE2 were noted to be absent in PBC as compared to controls.^47^ Comparable changes in PSC do not appear to occur as assessed by scRNA-seq of ECO. Indeed, we identified a DUOX2-positive population in ECO (data not shown); However, the expression of ACE2 was not observed in either PSC or non-PSC ECO derived from the large bile duct, and ACE2 expression appears to be limited to the intrahepatic cholangiocytes. This observation may reflect differences in the pathogenesis of the 2 diseases where PBC is an autoimmune-mediated ductopenic disease of intrahepatic cholangiocytes, whereas PSC is an inflammatory fibro-obliterative disease of extrahepatic and intrahepatic cholangiocytes.

It has previously been demonstrated that cholangiocytes express IL-17 receptors A and C and that they respond to IL-17A stimulation.^16^,^27^ However, the presence of the additional members of this receptor family and the differences in expression had yet to be characterized in human PSC cholangiocytes. In this study, we examined the expression of the IL-17 receptor subunits A–E in PSC and non-PSC patient-derived ECO. Our data suggest that IL-17RA, IL-17RC, and IL-17RE were equally expressed in both PSC and non-PSC ECO. Hence, differences in IL-17 receptor expression are unlikely to account for differences in IL-17 signaling between PSC and non-PSC ECO, or in PSC disease pathogenesis.

Once we confirmed that cholangiocytes express IL-17A receptors, we treated our PSC and non-PSC ECO with IL-17A and investigated differences in gene expression and protein secretion. Both PSC and non-PSC ECO responded to IL-17A with gene expression, indicating IL-17A signaling was intact. Some of the variations associated with PSC, in response to IL-17A, were identified in genes and proteins that display chemotactic activity for various immune cells, such as defensin beta 4A (DEFB4A), IL32, IL7, and CCL13. For instance, DEFB4A, acts as a ligand for CCR6 and induces chemotactic activity of CCR6-expressing cells, such as neutrophils, and has an important role in gastrointestinal host immune responses.^48^,^49^,^50^ Similarly, CXCL6 is known for its neutrophil chemotactic activity.^51^ On the other hand, IL32, CCL13, CXCL9, CXCL11, and IL7 have been associated with chemoattraction of activated T cells, and CCL25 has chemotactic activity on macrophages and dendritic cells.^52^,^53^,^54^,^55^ Collectively, these findings from diverse techniques, such as scRNA-seq, NanoString analysis, and secretome quantification, confirmed that the IL-17A signaling pathway is perturbed in PSC ECO. These alterations suggest that human PSC cholangiocytes respond to IL-17 by secreting chemokines, which, in turn, attract inflammatory cells to the bile duct, promoting further cellular inflammation and tissue damage.

However, unlike IBD,^36^ we did not identify rare somatic variants directly involving genes that are known to regulate the IL-17 signaling pathway. Nonetheless, rare somatic variants potentially regulating the MAPK signaling pathway were identified. Interestingly, the activation of the MAPK signaling pathway has been previously demonstrated to be a downstream effect of IL-17A stimulation;^56^ however, how these somatic gene alterations modulate specific IL-17 stimulation in cholangiocytes remains to be explored. We also speculate that there might be epigenetic changes in genes regulating the IL-17 signaling pathway, environmental factors, or effects caused by a combination of multiple polymorphisms driving this different response. Studies of the epigenome and the functional studies of the rare somatic variants potentially regulating the MAPK pathway are beyond the scope of this study but should be further examined.

Some of the limitations of this study are as follows: the control group, referred in this study as the non-PSC group, consists of patients who had a clinically indicated ERC. Therefore, they are not entirely healthy and the cholangiocyte microenvironment can also be that of inflammation. Additionally, given the rarity of PSC in the general population, this study includes a small sample size, which may overlook differences between our non-PSC and PSC conditions. However, despite these limitations, PSC-derived ECO still displays differences in IL-17A signaling, indicating that this signaling pathway is perturbed in these patients. Due to the extrahepatic origin of the ECO, this study does not permit a direct comparison between cholangiocytes from large versus those from small bile ducts, which may be important, given the diversity between cholangiocytes from different regions of the biliary tract. Cholangiocytes from small bile ducts would require their isolation from liver biopsies, which are rarely performed in this patient population. Also, the direct cell-to-cell interaction between cholangiocytes and IL-17 secreting cells needs to be examined to fully capture the effects of IL-17 on PSC cholangiocytes and replicate the paracrine signaling concentrations of IL-17 occurring in vivo. However, the development of mixed cellular ECO has yet to be described due to the inability of most cells to infiltrate the Matrigel matrix employed to culture ECO.

In conclusion, we examined the response of PSC versus non-PSC ECO to IL-17A stimulation. Although scRNA seq-based cluster analysis did not identify unique PSC clusters, IL-17A simulation uncovered differential gene expression and alterations in the secretome between the 2 patient populations. These observations are consistent with the concept that alteration of IL-17 signaling may contribute to the pathogenesis of PSC. These results also highlight the utility of ECO in examining disease mechanisms in PSC and suggest IL-17 directed therapy should be further explored in PSC.

## Supplementary Material

SUPPLEMENTARY MATERIAL

## Supplementary Material

SUPPLEMENTARY MATERIAL
