# Supplementary material for: IL-17 signaling in primary sclerosing cholangitis patient-derived organoids
Source: Hepatol Commun. 2024 Jun 3;8(6):e0454. doi: 10.1097/HC9.0000000000000454 (PMC11150034; doi:10.1097/HC9.0000000000000454)
Supplement: SUPPLEMENTARY MATERIAL [file hc9-8-e0454-s002.docx]

**Il-17 Signaling in Primary Sclerosing Cholangitis Patient-Derived Organoids**

Ana Sofia Garcia Moreno^1^, Maria Eugenia Guicciardi^1^, Alexander Q. Wixom^2^, Erik Jessen^2^, Jingchun Yang^1^, Sumera I. Ilyas^1^, Jackie K. Bianchi^1,^ Filippo Pinto e Vairo^3,4^, Konstantinos N. Lazaridis^1^, Gregory J. Gores^1^

**Supplemental Digital Content**

[**SDC, Materials and Methods 2**](#_Toc158816485)

[**SDC, Figure 1. 7**](#_Toc158816486)

[**SDC, Figure 2. 8**](#_Toc158816487)

[**SDC, Figure 3. 9**](#_Toc158816488)

[**SDC, Figure 4. 11**](#_Toc158816489)

[**SDC, Figure 5. 13**](#_Toc158816491)

[**SDC, Figure 6. 14**](#_Toc158816492)

[**SDC, Figure 7. 16**](#_Toc158816493)

[**SDC, Figure 8. 17**](#_Toc158816494)

[**SDC, Table. S1. 18**](#_Toc158816495)

[**SDC, Figure 9. 19**](#_Toc158816496)

[**SDC, Supplemental Excel files 20**](#_Toc158816497)

[**REFERENCES 20**](#_Toc158816498)

# SDC, Materials and Methods

**Materials.** Advanced Dulbecco’s modified Eagle’s medium/F12, Antibiotic-Antimycotic (Anti-Anti), and B-27 supplement were from Thermo Fisher Scientific (Waltham, MA); recombinant human R-spondin-1, recombinant human EGF, recombinant human HGF, recombinant human FGF10, and recombinant human noggin were from PeproTech (Cranbury, NJ):, gastrin, N-acetylcysteine, Y27632, nicotinamide and A83-01 were from Millipore-Sigma (Burlington, MA); human recombinant Wnt3A, Forskolin and human recombinant IL-17A were from R&D Systems (Minneapolis, MN). Matrigel (354230) and cell recovery solution were from Corning (Kennebunk, ME, USA).

**NanoString analysis.** ECO from PSC (n=6) and non-PSC patients (n=5) were treated with vehicle or recombinant human IL-17A (100 ng/mL) for 24 hours. Total RNA was extracted using RNeasy^®^ Plus mini kit (Qiagen, Hilden, Germany), and 100 ng of RNA was analyzed to determine the expression of a panel of immune-related genes using the nCounter_ Human Immunology V2 Panel (NanoString Technologies, Seattle, USA) according to the manufacturer’s instructions, employing an nCounter MAX analysis system. Functional annotation was then performed, by ingenuity pathway analysis (IPA) (available at www.qiagen.com/ingenuity), including only statistically significant differentially expressed genes (DEG).

**Single-cell RNA-seq analysis.** ECO from PSC (n=4) and non-PSC patients (n=4), all at passage 3, were treated with vehicle or human recombinant IL-17A (100 ng/mL) for 24 hours. The organoids underwent trypsin digestion followed by mechanical dissociation to obtain single cell suspensions in PBS + 0.1% BSA and were submitted to the Gene Analysis Core of the Medical Genome Facility (Mayo Clinic, Rochester, MN) for scRNA-seq analysis. Cell number and viability were measured using the Vi-Cell XR Cell Viability Analyzer (Beckman-Coulter, Brea, CA). The cDNA master mix was prepared according to the manufacture’s instruction for Chromium Next GEM Single Cell 3’ Library and Gel Bead Kit (10x Genomics, Pleasanton, CA). The standard targeted cell recovery was set to ~3000 cells. All cDNA pools and resulting libraries were measured using Qubit High Sensitivity assays (Thermo Fisher Scientific, Waltham, MA) and Agilent Bioanalyzer High Sensitivity chips (Agilent, Santa Clara, CA). Libraries were sequenced at between 40,000 and 50,000 fragment reads per cell following Illumina’s standard protocol using the Illumina cBot and HiSeq 3000/4000 PE Cluster Kit (Illumina, San Diego, CA). The flow cells were sequenced as 100 X 2 paired end reads on an Illumina HiSeq 4000 HD using HiSeq 3000/4000 sequencing kit and HCS v3.4.0.38 collection software. Base-calling was performed using Illumina’s RTA version 2.7.7.

After sequencing, the ECO samples were processed using 10x Genomics Cell Ranger 5.0.0 ^(1)^. Each sample’s aligned reads were then imported into R for quality control processing by Seurat^(2)^ (v4), filtering cells with less than 200 nCounts or greater than 40% mitochondrial reads. All samples were then normalized by SCTransform (v2) and integrated using Harmony (v0.1.1)^(3)^. Analyses were performed following Seurat recommendations. Filtering of marker differentially expressed genes was performed using a q-value < 0.05, an |log_2-_fold change| > 0.2, and a custom parameter: percent expression change ratio > 0.2 (when comparing treatments or conditions). The percent expression change ratio was calculated using the percent of cells showing expression of the feature in the groups being compared (pct.1/pct.2 or pct.2/pct1 with the greater being the numerator). Code for all analyses is available upon request.

**Organoid immunofluorescence.** ECO were grown in 30-μl matrigel domes in 35 mm glass bottom Petri dishes (GBD00004-200; Cell E&G LLC, San Diego, CA). Whole mount immunofluorescence for cytokeratin 7 (KRT7) and SRY-Box transcription factor 9 (SOX9), both cholangiocyte markers, was performed as previously described.^(4)^ Mouse monoclonal anti-KRT7 antibody (#sc-400628, Santa Cruz Biotechnology, Santa Cruz, CA) and rabbit monoclonal anti-SOX9 antibody (D8G8H, #82630, Cell Signaling, Danvers, MA) were diluted 1:100. Secondary antibodies, goat anti-mouse IgG AlexaFluor488 and chicken anti-rabbit IgG AlexaFluor594 (Invitrogen/Thermo Fisher Scientific) were used for KRT7 and SOX9, respectively, at a 1:200 dilution. 4′,6-diamidino-2-phenylindole (DAPI, 300 nM) was added together with the secondary antibodies to visualize the nuclei. The slides were mounted with ProLong™ Gold Antifade Mountant (Thermo Fisher Scientific) and analyzed by confocal microscopy using ZEISS LSM 980 (Zeiss, Oberkochen, Germany).

**Genome sequencing (GS).** ECO from 9 PSC patients were subjected to GS. DNA isolation was performed using the QIAamp Blood Mini Kit (Qiagen). Samples were submitted to the Gene Analysis Core of the Medical Genome Facility (Mayo Clinic, Rochester, MN) for GS. Libraries were prepared using up to 500 ng genomic DNA according to the manufacturer’s instructions for the Nextera DNA Flex Library Prep Kit (Illumina, San Diego, CA). The concentration and size distribution of the completed libraries were determined using the Fragment Analyzer (Agilent, Santa Clara, CA) and Qubit fluorometry (Invitrogen, Carlsbad, CA). Libraries were sequenced at an average coverage of approximately 40x following Illumina's standard protocol for the Illumina NovaSeq 6000 and S2 flow cell. The flow cells were sequenced as 150 x 2 paired end reads using the NovaSeq S2 v1.5 sequencing kit and NovaSeq Control Software v1.7.0. Base-calling was performed using Illumina’s RTA version 3.4.4.

To differentiate between somatic and germline variants, the results of the GS were compared to the data obtained from previous ES on the patient’s peripheral white blood cell (PWBC) samples. Isolation of genomic DNA from blood samples was performed by the Biospecimens Accessioning and Processing laboratory at the Mayo Clinic using the PureGene kit (Gentra Systems, Minneapolis, MN, USA) as specimens were received and submitted to the Gene Analysis Core of the Medical Genome Facility (Mayo Clinic, Rochester, MN) for ES. Paired-end libraries were prepared with approximately 400 ng of genomic DNA using the SureSelect XT Low Input Reagent Kit (Agilent, Santa Clara, CA). The concentration and size distribution of the completed libraries were determined using an Agilent Bioanalyzer DNA 1000 chip or Advance Fragment Analyzer and Qubit fluorometry (Invitrogen, Carlsbad, CA). Adaptor-ligated DNA was amplified with the SureSelect Post-Capture forward and specific index reverse primers for 12 cycles. Exon capture was conducted using 750 ng of the prepared library following the protocol for Agilent’s SureSelect Human All Exon v5 + UTRs 75 MB kit. The concentration and size distribution of the completed captured libraries were determined on Qubit (Invitrogen) and an Agilent Bioanalyzer DNA 1000 chip. Libraries were sequenced, yielding approximately 43 million to 59 million read pairs per sample, following Illumina's standard protocol for the Illumina NextSeq 2000. The NextSeq P2 flow cell was sequenced as 150 x 2 paired end reads using the NextSeq 1000/2000 Control Software v1.4.1 and RTA3.

**Somatic variant analysis.** Somatic variants were called using both Mutect2^(5)^ and Strelka^(6)^ variant callers. The reference samples were exome sequenced bam files and the somatic were genome sequenced bam files, both aligned to the hg38 human genome. Default parameters were used for both tools. Somatic variant calls were filtered on the ‘PASS’ flag from Mutect2 and Strelka variant callers, a read depth of 10 reads in both the GS tumor and ES normal/reference sample. Somatic variants were further annotated using BioR^(7)^ variant annotation with information from CAVA^(8)^ and 1000 genomes^(9)^, ExAC^(10)^, and gnomAD^(11)^ databases. Variants with a population allele frequency of less than 0.0001 were kept as rare variants. Any variant with a ‘likely artifact’ designation in gnomAD was removed. Finally, variants were annotated with a CADD score^(12)^ to quantitate the predicted impact of the variant, keeping variants with a CADD score greater than 25 (Fig.S7.). CADD was chosen over other prediction tools because it integrates multiple annotations into one metric and includes splicing scores and annotations related to non-coding regions of the genome. Relaxed variants were obtained by excluding impact factor and CADD score from filtering criteria (Fig.S7) Relaxed variants were input into Cytoscape^(13)^ for pathway enrichment and visualization using Enrichment Map and AutoAnnotate. KEGG^(14)^ and GO pathways with a p-value <0.05 were selected for plotting.

**Secretome analysis by Olink analysis.** ECO from PSC (n=5) and non-PSC (n=7) patients at passage 4-6 were treated with IL-17A (100 ng/ml) or vehicle in expansion medium. After 24 hr., medium was removed, centrifuged at 1,500 x g for 5 min and the supernatants stored at -80°C until used. In addition, organoid expansion medium exposed to matrigel without organoids for 24 hr. was used as negative control. The organoids were collected, centrifuged at 300 x g for 5 min, and lysed in RIPA buffer (50 mM TRIS-HCl, pH 7.4, 1% NP-40, 0.25% NaDCA, 1 mM EDTA, 1 mM Na_3_VO_4_, 1 mM NaF, protease inhibitor mix). Protein content was measured by BCA assay (Thermo Fisher) and used as normalization factor. ECO supernatants were plated in a randomized fashion and shipped to the University of Minnesota Genomic Center (Oakdale, MN) where they were analyzed by proximity extension technology (Olink, Uppsala, Sweden) using the Olink^®^ Target 96 Inflammation panel. Data were expressed as normalized protein expression (NPX) values. To account for the variability in organoid number across samples, and the proteins contained in the medium and matrigel, adjusted NPX values were calculated by subtracting the corresponding NPX value from the negative control (baseline) for each protein and normalizing to the -Log2 of the organoid protein content.

# SDC, Figure 1. Identification of distinct cholangiocyte cluster populations in ECO by scRNA seq.


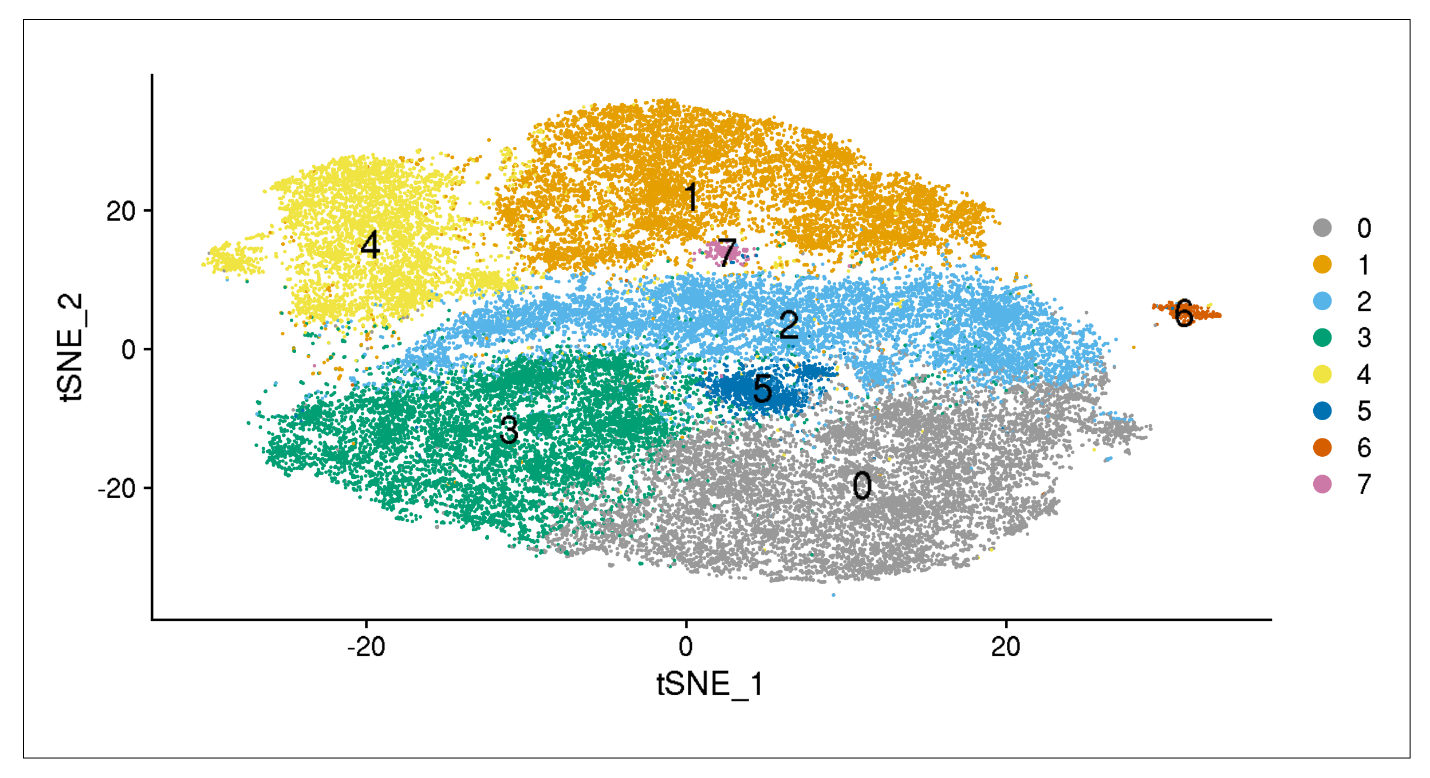


**Fig. S1**. Identification of distinct cholangiocyte cluster populations in ECO by scRNA seq. TSNE plot displaying distinct clusters termed Cluster (0) to (8), obtained by unsupervised clustering analysis from patient-derived ECO. This demonstrates an adequate clustering of cells into each respective cluster population. Colors represent a unique cell population cluster as identified by transcriptional signature.

# SDC, **Figure 2.** Cholangiocyte cluster population’s quantitative differences between non-PSC and PSC ECO after stimulation with vehicle or IL-17A.


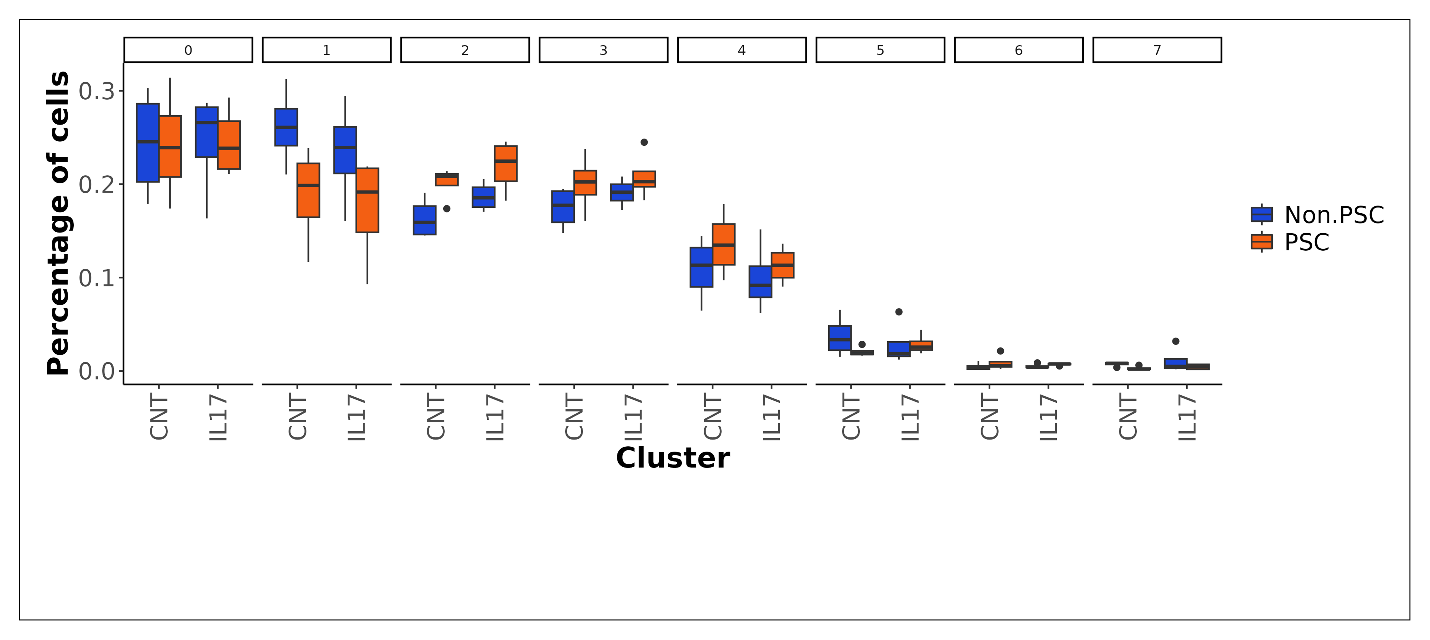


**Fig. S2.** Cholangiocyte cluster population’s quantitative differences between non-PSC and PSC ECO after stimulation with vehicle or IL-17A. Box plot visualizing Cluster’s (0) to (8) quantitative changes on cell percentage in response to vehicle and IL-17A treatment. No significant differences (p<0.05) were found at baseline or after IL-17A stimulation at a concentration of 100 ng/mL for 24 hours.


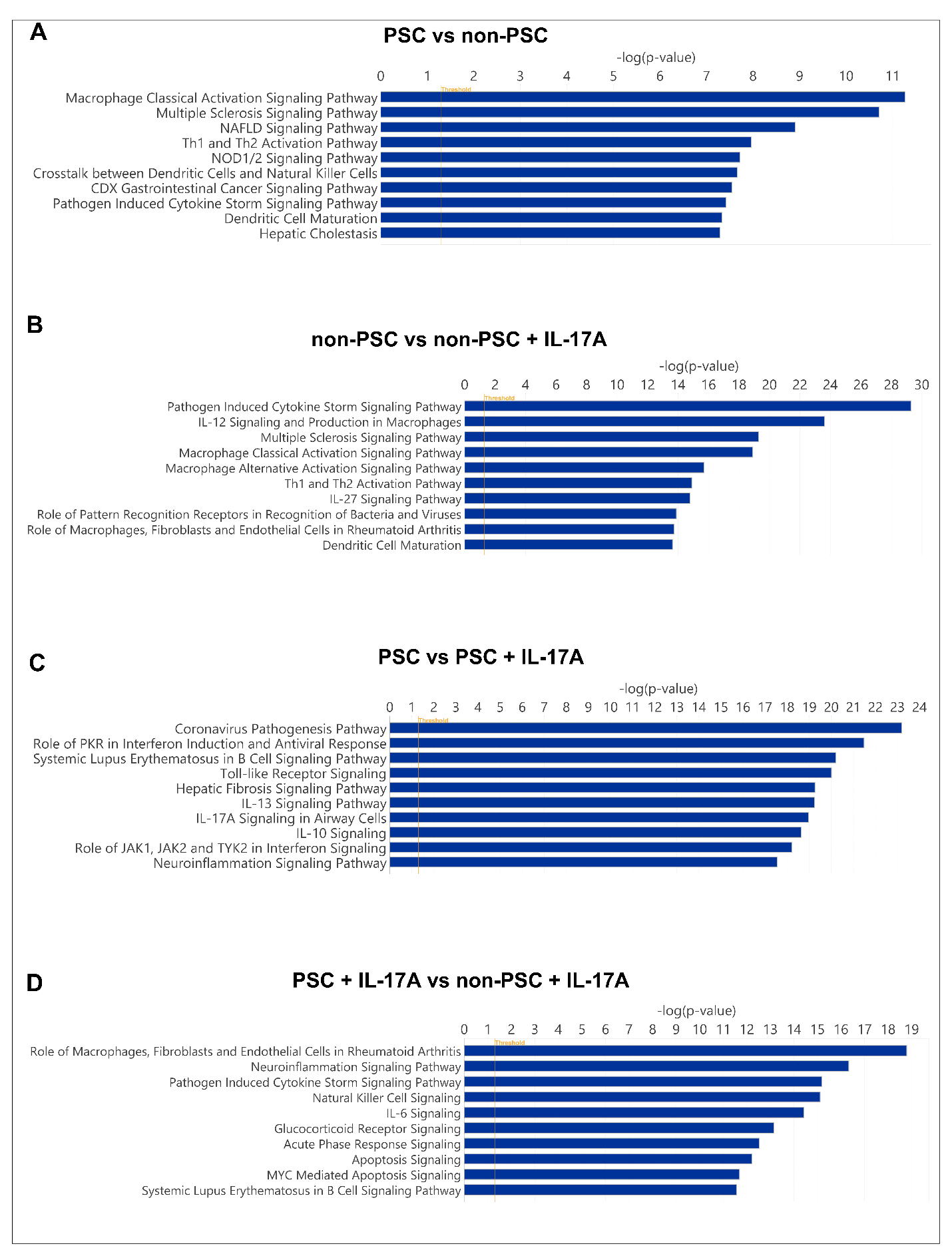
SDC, Figure 3. Functional annotation of differentially expressed genes (DEG) between non-PSC and PSC ECO using ingenuity pathway analysis (IPA).

**Fig. S3.** Functional annotation of differentially expressed genes (DEG) between non-PSC and PSC ECO using ingenuity pathway analysis (IPA). Differential gene expression analysis was conducted using NanoString, and the identified DEG were subjected to Ingenuity Pathway Analysis (IPA) available at www.qiagen.com/ingenuity. The top 10 disease and function annotations of DEGs were visualized using bar charts. A) Comparison between non-PSC and PSC ECO. B) Comparison between non-PSC ECO treated with vehicle and non-PSC ECO after stimulation with IL-17A. C) Comparison between PSC ECO treated with vehicle and PSC ECO after stimulation with IL-17A. D) Comparison between non-PSC and PSC ECO after stimulation with IL-17A.


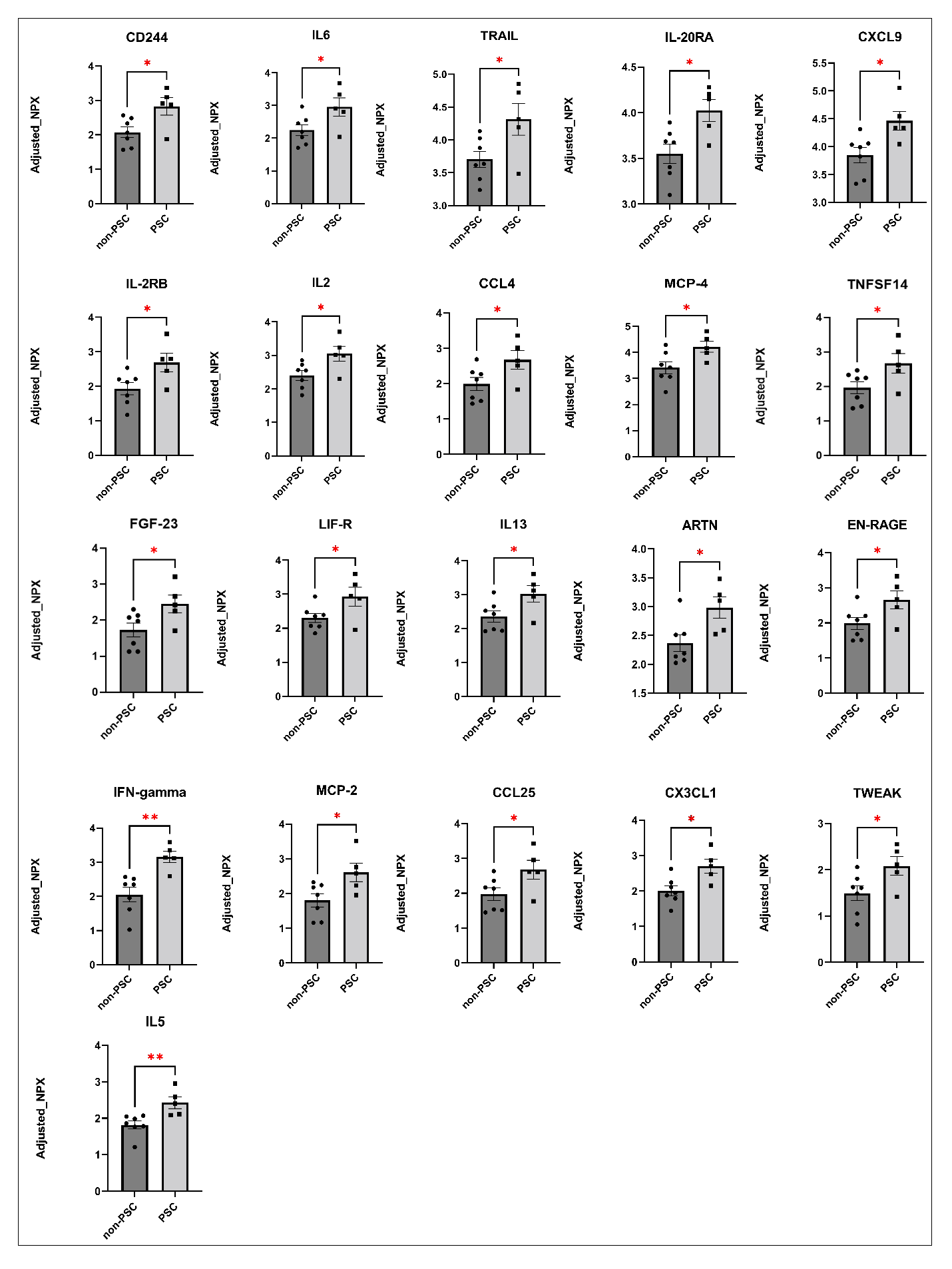
SDC, Figure 4. Significantly upregulated inflammation-related proteins in PSC vs. non-PSC ECO supernatant.

Fig. S4. Significantly upregulated inflammation-related proteins in PSC vs. non-PSC ECO supernatant. Differential abundance of inflammatory proteins in PSC vs. non-PSC ECO supernatant determined by Olink analysis expressed as adjusted NPX values. (*p < 0.05, **p < 0.01)

SDC, Figure 5. Expression of genes associated with cholangiocyte driven fibrosis.


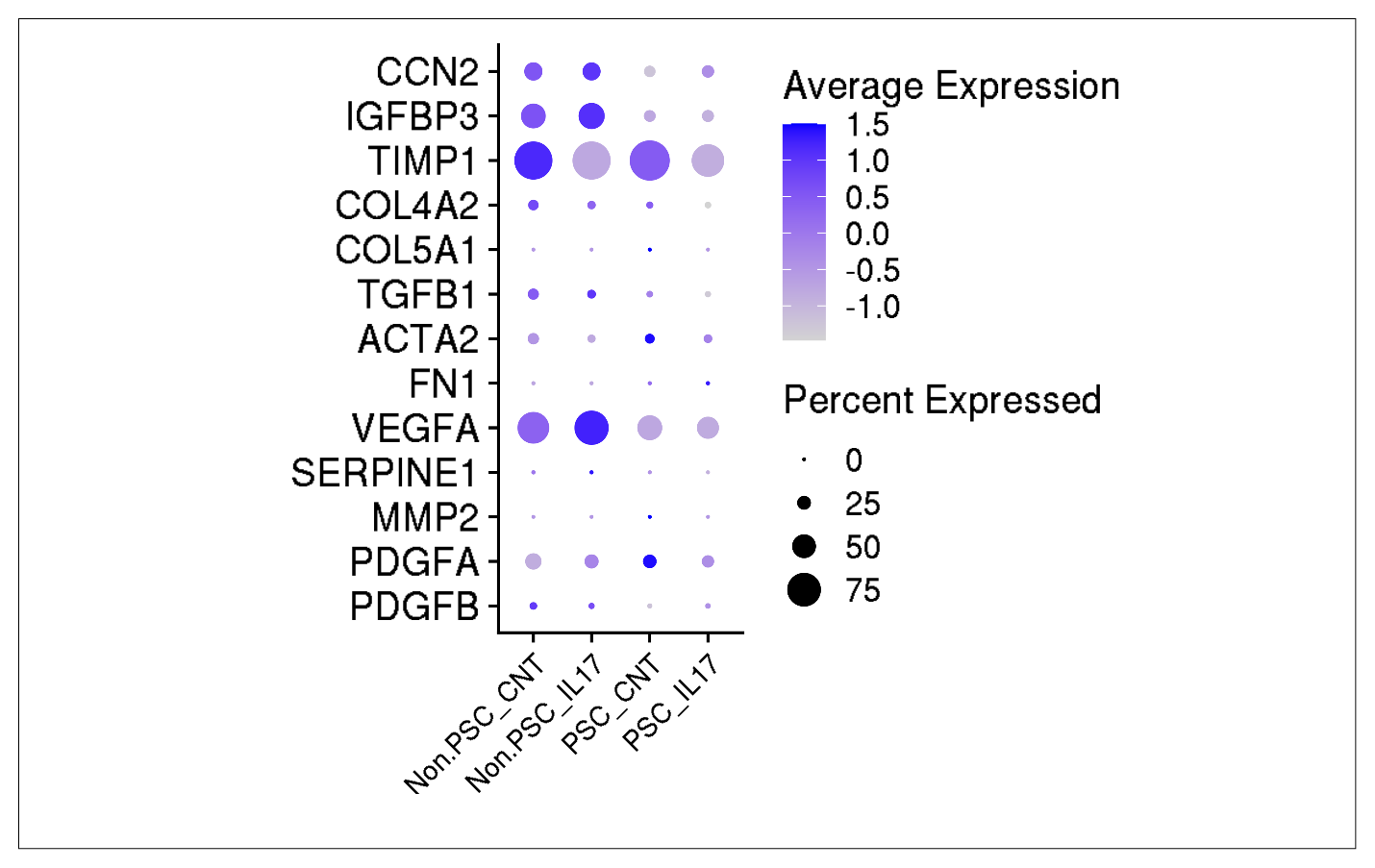


**Fig. S5.** Expression of genes associated with cholangiocyte driven fibrosis. Dot plot displaying expression of known markers of cholangiocyte driven fibrosis in PSC and non-PSC, and treatments (vehicle and IL-17A) by scRNA-seq analysis of ECO. The intensity of the color indicates the average expression, and the size of the circle is directly proportional to the percentage of cells expressing each gene. No significant differences were identified between the groups. (p<0.05).


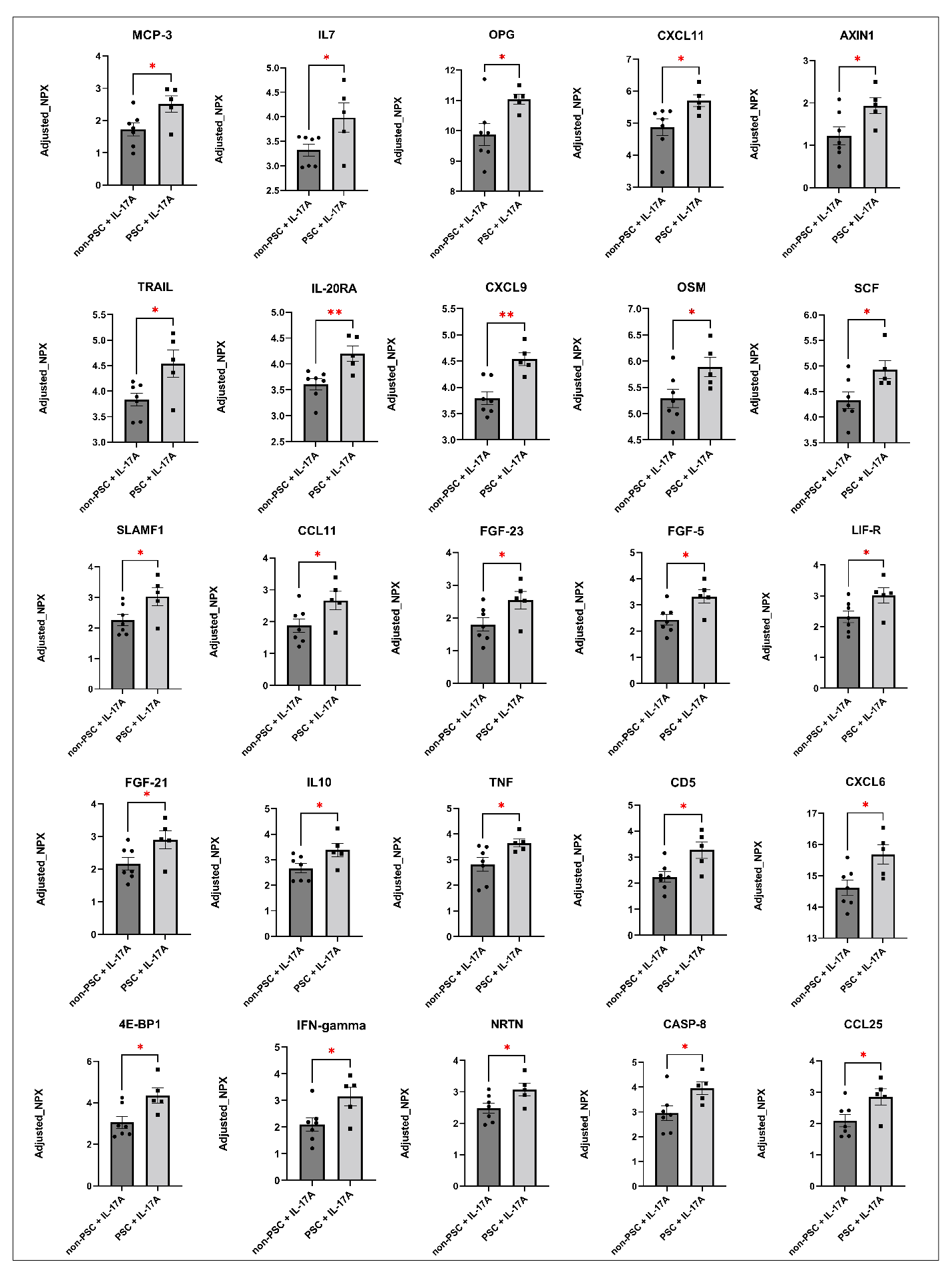
SDC, Figure 6. Significantly upregulated inflammation-related proteins in PSC vs. non-PSC ECO supernatant after IL-17A stimulation.


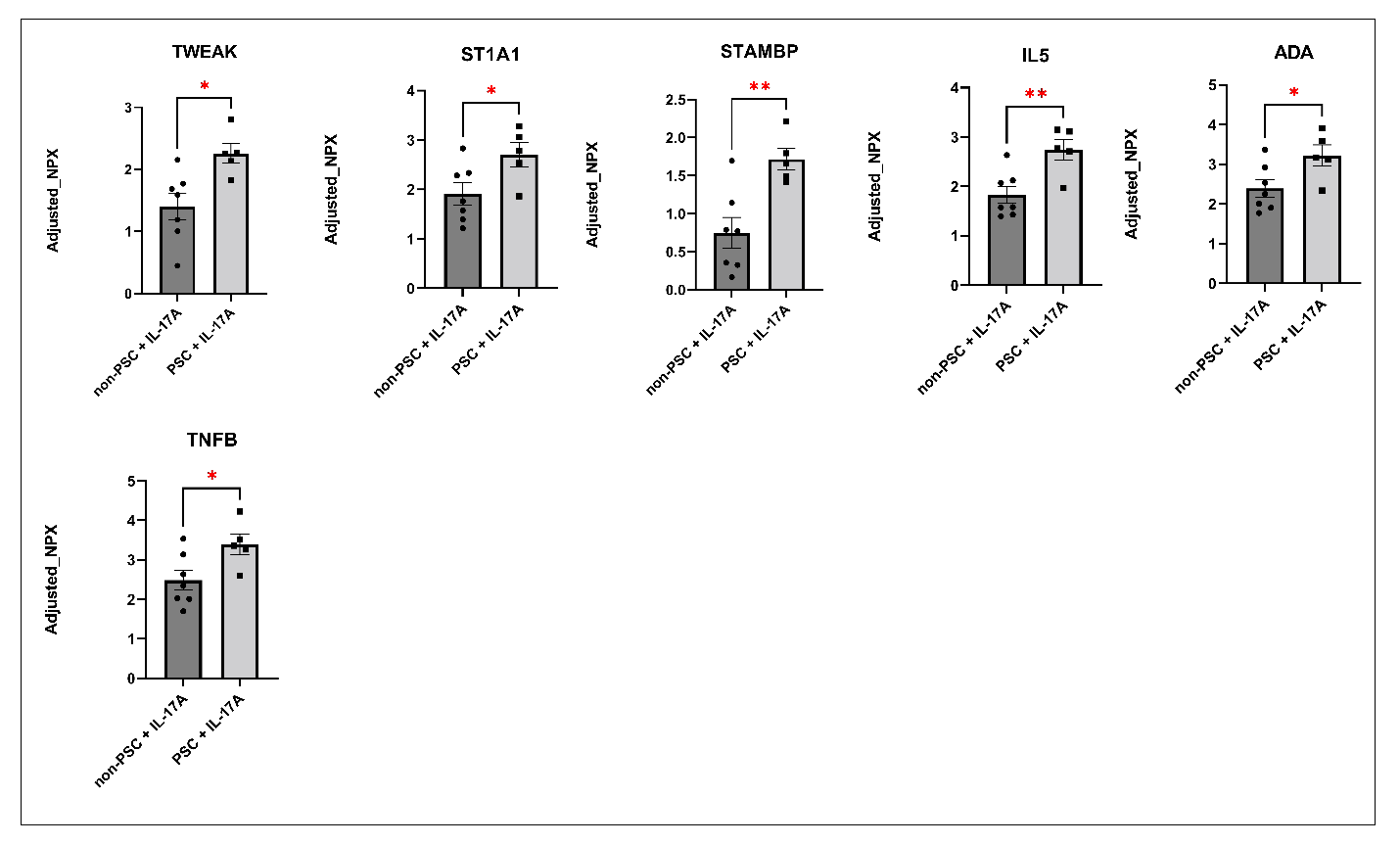


**Fig. S6.** Significantly upregulated inflammation-related proteins in PSC vs. non-PSC ECO supernatant after IL-17A stimulation. Differential abundance of inflammatory proteins in PSC vs. non-PSC ECO supernatant after IL-17A stimulation determined by Olink analysis expressed as adjusted NPX values. (*p < 0.05, **p < 0.01).

# SDC, Figure 7. Methodology employed for somatic variant identification in PSC ECO.


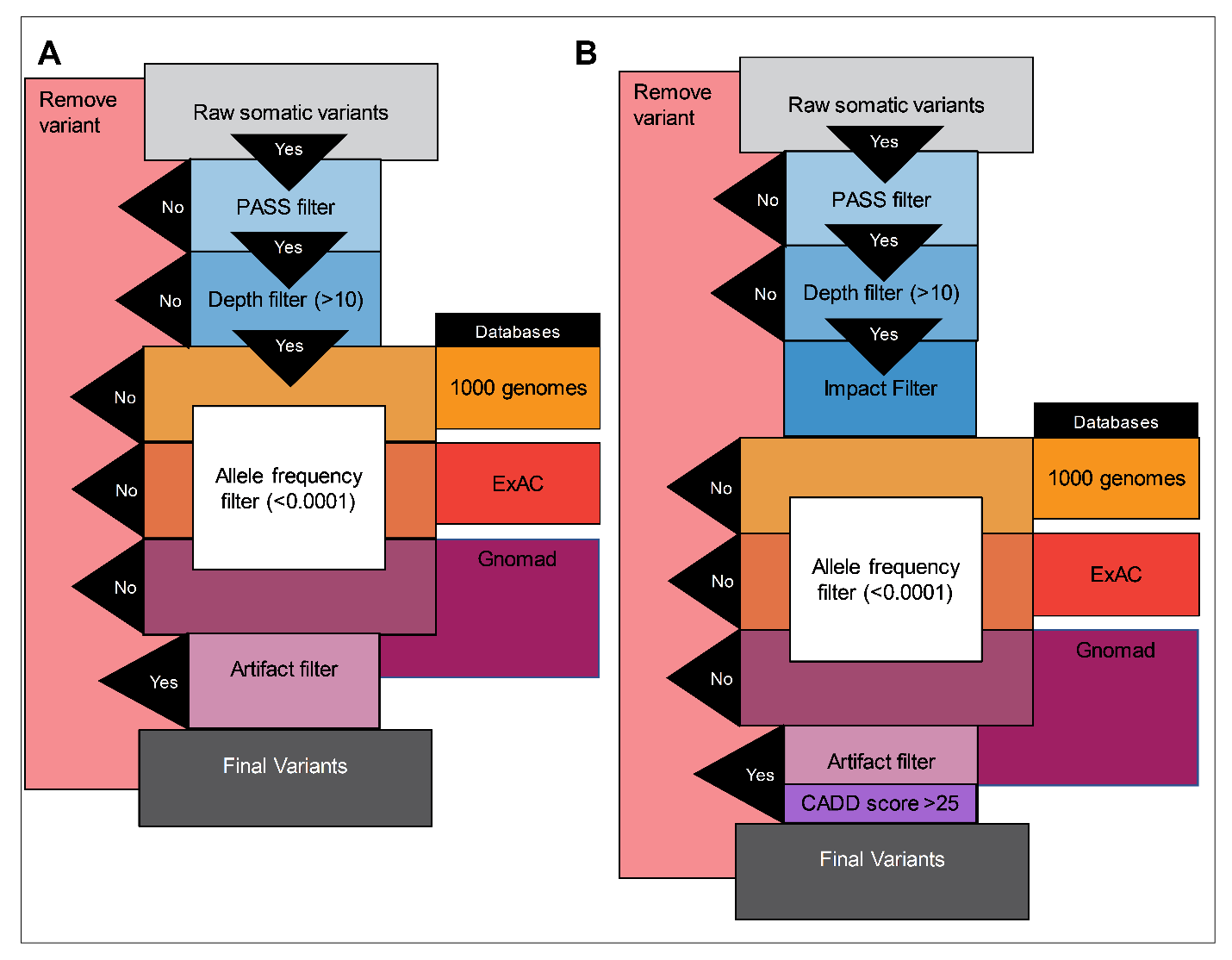


**Fig. S7.** Methodology employed for somatic variant identification in PSC ECO. A) Diagram visualizing the filtering criteria employed for the identification of somatic variants with a CADD score >25. B) Diagram visualizing the filtering criteria employed for the identification of relaxed somatic variants.

# SDC, Figure 8. KEGG pathway map for human MAPK signaling pathway.


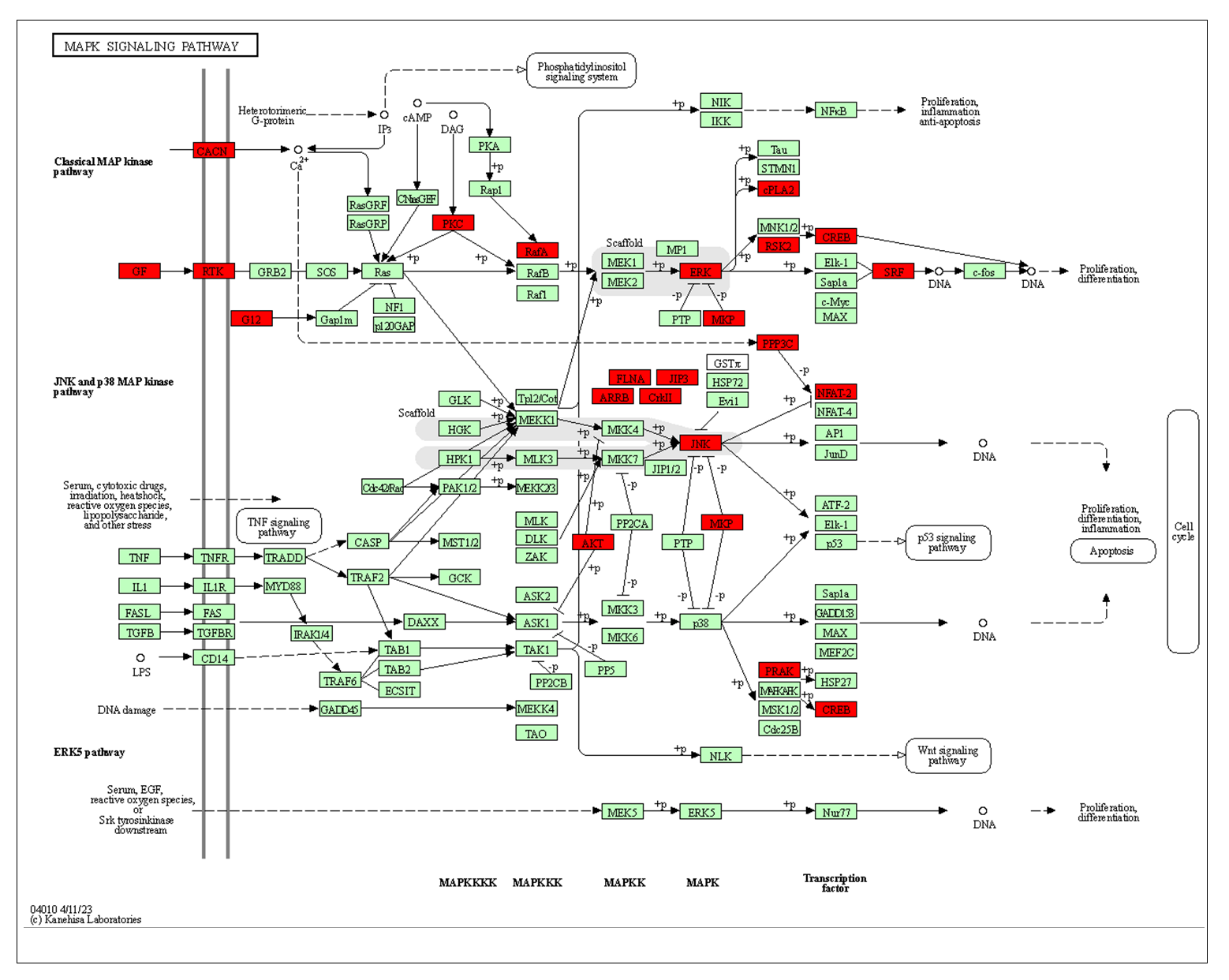


**Fig. S8.** KEGG pathway map for human MAPK signaling pathway. Diagram visualizing genes with rare somatic variants present in PSC ECO (red) within the MAPK signaling pathway by KEGG pathway analysis^[(14)].^

# SDC, Table. S1. Rare somatic variants in genes potentially regulating the MAPK signaling pathway.


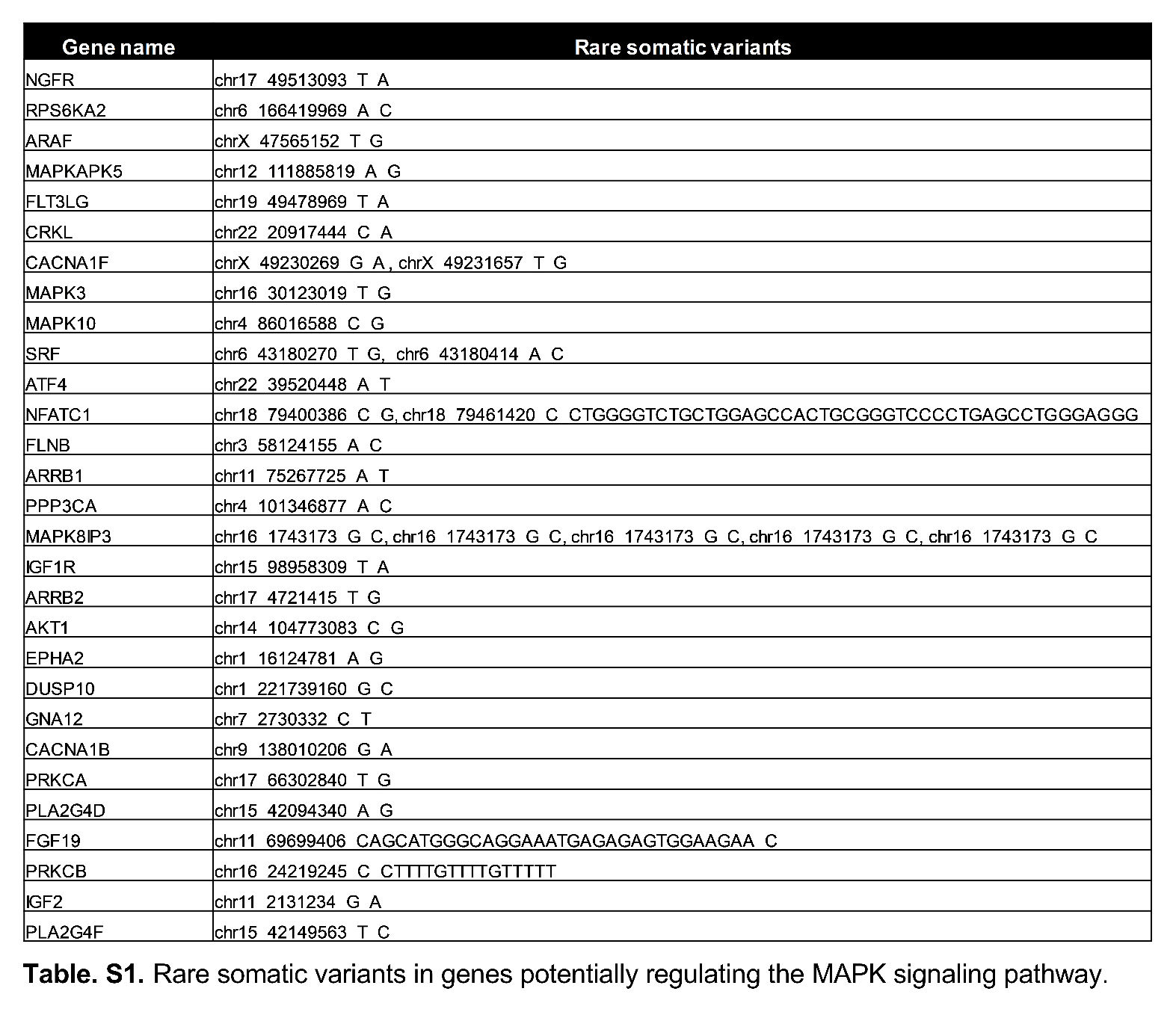


# SDC, Figure 9. PSC and non-PSC ECO respond differently to IL-17A stimulation as assessed by scRNA-seq, NanoString and secretome analysis.

**
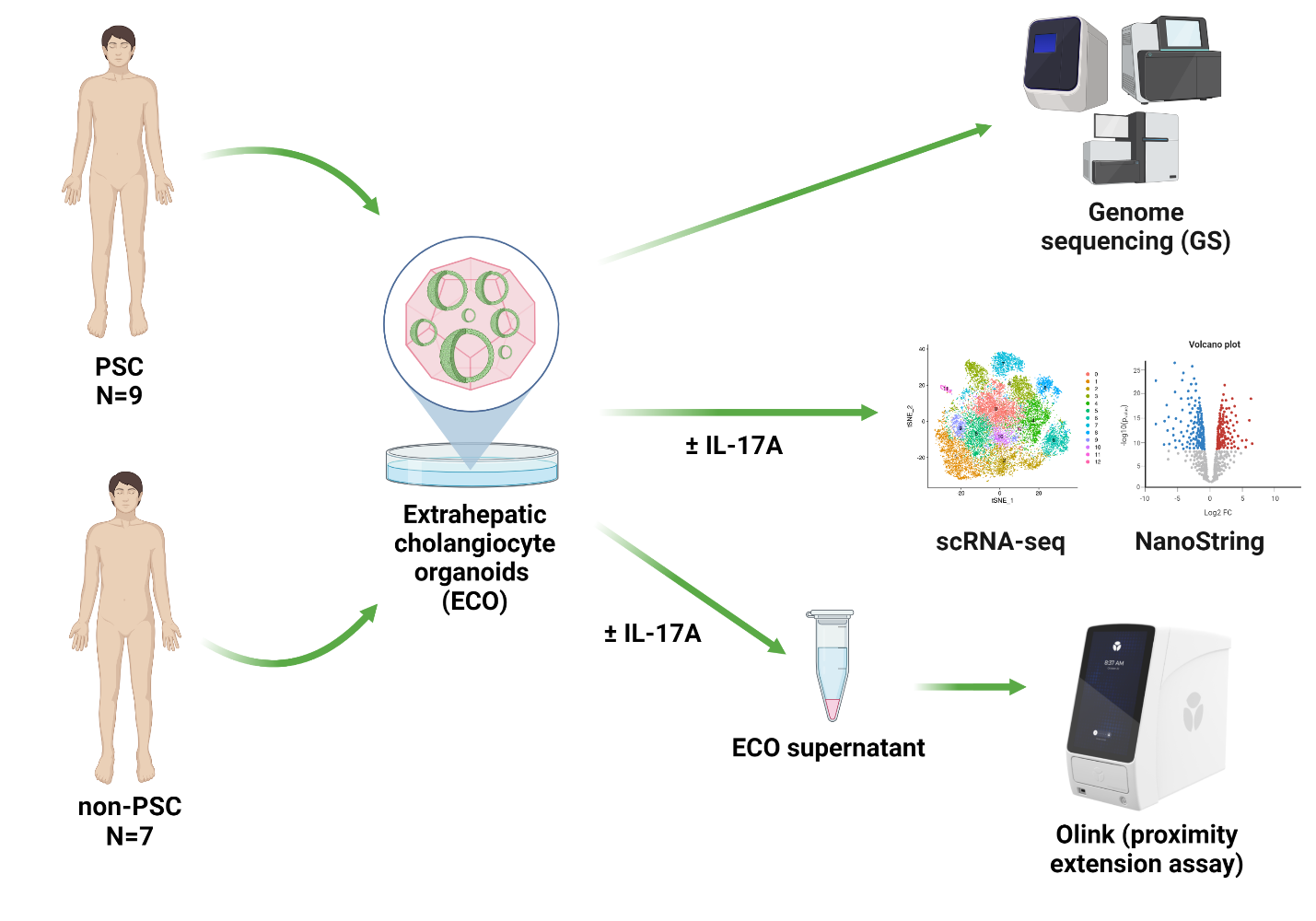
**

# SDC, Supplemental Excel files:

- Supplementary file 1. (Conserved cluster marker genes).
- Supplementary file 2. DEG PSC vs non-PSC treated with vehicle.
- Supplementary file 3. DEG PSC vs non-PSC treated with IL-17A.
- Supplementary file 4. DEG non-PSC vehicle vs non-PSC IL-17A.
- Supplementary file 5. DEG PSC vehicle vs PSC IL-17A.

# REFERENCES

1. Zheng GX, Terry JM, Belgrader P, Ryvkin P, Bent ZW, Wilson R, Ziraldo SB, et al. Massively parallel digital transcriptional profiling of single cells. Nat Commun 2017;8:14049.

2. Hao Y, Hao S, Andersen-Nissen E, Mauck WM, 3rd, Zheng S, Butler A, Lee MJ, et al. Integrated analysis of multimodal single-cell data. Cell 2021;184:3573-3587.e3529.

3. Korsunsky I, Millard N, Fan J, Slowikowski K, Zhang F, Wei K, Baglaenko Y, et al. Fast, sensitive and accurate integration of single-cell data with Harmony. Nat Methods 2019;16:1289-1296.

4. Rezanejad H, Lock JH, Sullivan BA, Bonner-Weir S. Generation of Pancreatic Ductal Organoids and Whole-Mount Immunostaining of Intact Organoids. Curr Protoc Cell Biol 2019;83:e82.

5. Geraldine A. Van der Auwera BDOC. Genomics in the Cloud. 1st ed: O'Reilly Media, Inc., 2020.

6. Kim S, Scheffler K, Halpern AL, Bekritsky MA, Noh E, Källberg M, Chen X, et al. Strelka2: fast and accurate calling of germline and somatic variants. Nat Methods 2018;15:591-594.

7. Kocher J-PA, Quest DJ, Duffy P, Meiners MA, Moore RM, Rider D, Hossain A, et al. The Biological Reference Repository (BioR): a rapid and flexible system for genomics annotation. Bioinformatics 2014;30:1920-1922.

8. Münz M, Ruark E, Renwick A, Ramsay E, Clarke M, Mahamdallie S, Cloke V, et al. CSN and CAVA: variant annotation tools for rapid, robust next-generation sequencing analysis in the clinical setting. Genome Medicine 2015;7:76.

9. Fairley S, Lowy-Gallego E, Perry E, Flicek P. The International Genome Sample Resource (IGSR) collection of open human genomic variation resources. Nucleic Acids Research 2019;48:D941-D947.

10. Lek M, Karczewski KJ, Minikel EV, Samocha KE, Banks E, Fennell T, O’Donnell-Luria AH, et al. Analysis of protein-coding genetic variation in 60,706 humans. Nature 2016;536:285-291.

11. Chen S, Francioli LC, Goodrich JK, Collins RL, Kanai M, Wang Q, Alföldi J, et al. A genome-wide mutational constraint map quantified from variation in 76,156 human genomes. bioRxiv 2022:2022.2003.2020.485034.

12. Rentzsch P, Witten D, Cooper GM, Shendure J, Kircher M. CADD: predicting the deleteriousness of variants throughout the human genome. Nucleic Acids Research 2018;47:D886-D894.

13. Shannon P, Markiel A, Ozier O, Baliga NS, Wang JT, Ramage D, Amin N, et al. Cytoscape: a software environment for integrated models of biomolecular interaction networks. Genome Res 2003;13:2498-2504.

14. Kanehisa M, Furumichi M, Sato Y, Kawashima M, Ishiguro-Watanabe M. KEGG for taxonomy-based analysis of pathways and genomes. Nucleic Acids Res 2023;51:D587-d592.
